# Single-cell RNA sequencing of subcutaneous adipose tissues identifies therapeutic targets for cancer-associated lymphedema

**DOI:** 10.1038/s41421-022-00402-5

**Published:** 2022-06-21

**Authors:** Xuanyu Liu, Meng Yuan, Qinqin Xiang, Zhujun Li, Fen Xu, Wen Chen, Jie Chen, Jiuzuo Huang, Nanze Yu, Zhou Zhou, Xiao Long

**Affiliations:** 1 State Key Laboratory of Cardiovascular Disease, Beijing Key Laboratory for Molecular Diagnostics of Cardiovascular Diseases, Center of Laboratory Medicine, Fuwai Hospital, National Center for Cardiovascular Diseases, Chinese Academy of Medical Sciences and Peking Union Medical College, Beijing, China; 2 Prenatal Diagnosis Center, Department of Obstetrics and Gynecologic, Key Laboratory of Birth Defects and Related Diseases of Women and Children, Ministry of Education, West China Second University Hospital, Sichuan University, Chengdu, Sicuan, China; 3 Division of Plastic Surgery, Peking Union Medical College Hospital, Beijing, China

**Keywords:** Mechanisms of disease, Cervical cancer

## Abstract

Cancer-associated lymphedema frequently occurs following lymph node resection for cancer treatment. However, we still lack effective targeted medical therapies for the treatment or prevention of this complication. An in-depth elucidation of the cellular alterations in subcutaneous adipose tissues of lymphedema is essential for medical development. We performed single-cell RNA sequencing of 70,209 cells of the stromal vascular fraction of adipose tissues from lymphedema patients and healthy donors. Four subpopulations of adipose-derived stromal cells (ASCs) were identified. Among them, the *PRG4*^+^/*CLEC3B*^+^ ASC subpopulation c3 was significantly expanded in lymphedema and related to adipose tissue fibrosis. Knockdown of *CLEC3B* in vitro could significantly attenuate the fibrogenesis of ASCs from patients. Adipose tissues of lymphedema displayed a striking depletion of *LYVE*^+^ anti-inflammatory macrophages and exhibited a pro-inflammatory microenvironment. Pharmacological blockage of Trem1, an immune receptor predominantly expressed by the pro-inflammatory macrophages, using murine LR12, a dodecapeptide, could significantly alleviate lymphedema in a mouse tail model. Cell–cell communication analysis uncovered a perivascular ligand-receptor interaction module among ASCs, macrophages, and vascular endothelial cells. We provided a comprehensive analysis of the lineage–specific changes in the adipose tissues from lymphedema patients at a single-cell resolution. *CLEC3B* was found to be a potential target for alleviating adipose tissue fibrosis. Pharmacological blockage of TREM1 using LR12 could serve as a promising medical therapy for treating lymphedema.

## Introduction

Lymphedema is defined by chronic tissue edema that results from lymphatic drainage disorders due to intrinsic fault (primary lymphedema) or damage (secondary lymphedema) to the lymphatic system^1^. Secondary lymphedema is the most prevalent form and frequently occurs following lymph node resection for cancer treatment, i.e., cancer-associated lymphedema^2^. Up to 20% of women develop this condition following treatment for breast cancer^3^. Lymphedema is characterized by progressive swelling, chronic inflammation, excessive fibrosis, and adipose deposition in the affected limbs^4^. Lymphedema usually exerts a significant physical and psychological burden on cancer survivors and severely affects their quality of life; however, the clinical treatment remains palliative^5^. We still lack effective therapies, in particular, targeted medical therapies, for the treatment or prevention of this complication, which is partially due to the incomplete understanding of the cellular mechanisms underlying the pathogenesis.

Adipose tissue is not simply a container of fat, but an endocrine organ, which is composed of multiple types of cells, such as adipose-derived stromal cells (ASCs), adipocytes, vascular cells (e.g., vascular endothelial cells and pericytes), and immune cells^6^. All nonadipocyte cells are known as the stromal vascular fraction (SVF), which can be isolated through enzymatic digestion^7^. Lymphatic fluid stasis in the limbs of patients with lymphedema will ultimately result in increased subcutaneous adipose tissue volume and excess adipose deposition, which may lead to further deterioration of the lymphatic system^8^. Previous studies have reported significant alterations in the SVF of subcutaneous adipose tissues in lymphedema concerning cellular composition, proliferation, and differentiation capacity, and these studies highlight the important role of the SVF alterations in the pathophysiology of lymphedema^9–12^. However, previous studies generally relied on the expression of a limited number of markers and focused on a few cell lineages. We still lack a comprehensive and accurate understanding of the cellular and molecular alterations in subcutaneous adipose tissues of lymphedema.

Recent technical advances in single-cell RNA sequencing (scRNA-seq) have enabled the transcriptomes of tens of thousands of cells to be assayed at a single experiment^13^. Compared with bulk RNA-seq, scRNA-seq allows for unbiased cellular heterogeneity dissection at an unprecedented resolution^14^. scRNA-seq is emerging as a powerful tool for understanding the cellular and molecular mechanisms underlying the pathogenesis of diseases such as pulmonary fibrosis^15^ and lupus nephritis^16^. scRNA-seq has been applied to dissect the cellular heterogeneity of the SVF in mice^17,18^ and humans^6^ in healthy conditions. However, to our knowledge, few studies have been performed to explore the alterations in the SVF under a diseased condition, for example, lymphedema, at a single-cell resolution.

In this study, we performed scRNA-seq of 70,209 cells of the SVF of subcutaneous adipose tissues from patients with cancer-associated lymphedema and healthy donors. We sought to identify the cellular subpopulations associated with the disease, lineage-specific regulatory changes, and intercellular communication alterations in adipose tissues of lymphedema. The ultimate purpose of this study was to find novel medical targets or therapies for the prevention or treatment of lymphedema.

## Results

### scRNA-seq reveals cellular diversity and heterogeneity of subcutaneous adipose tissues in cancer-associated lymphedema

**Fig. 1:**
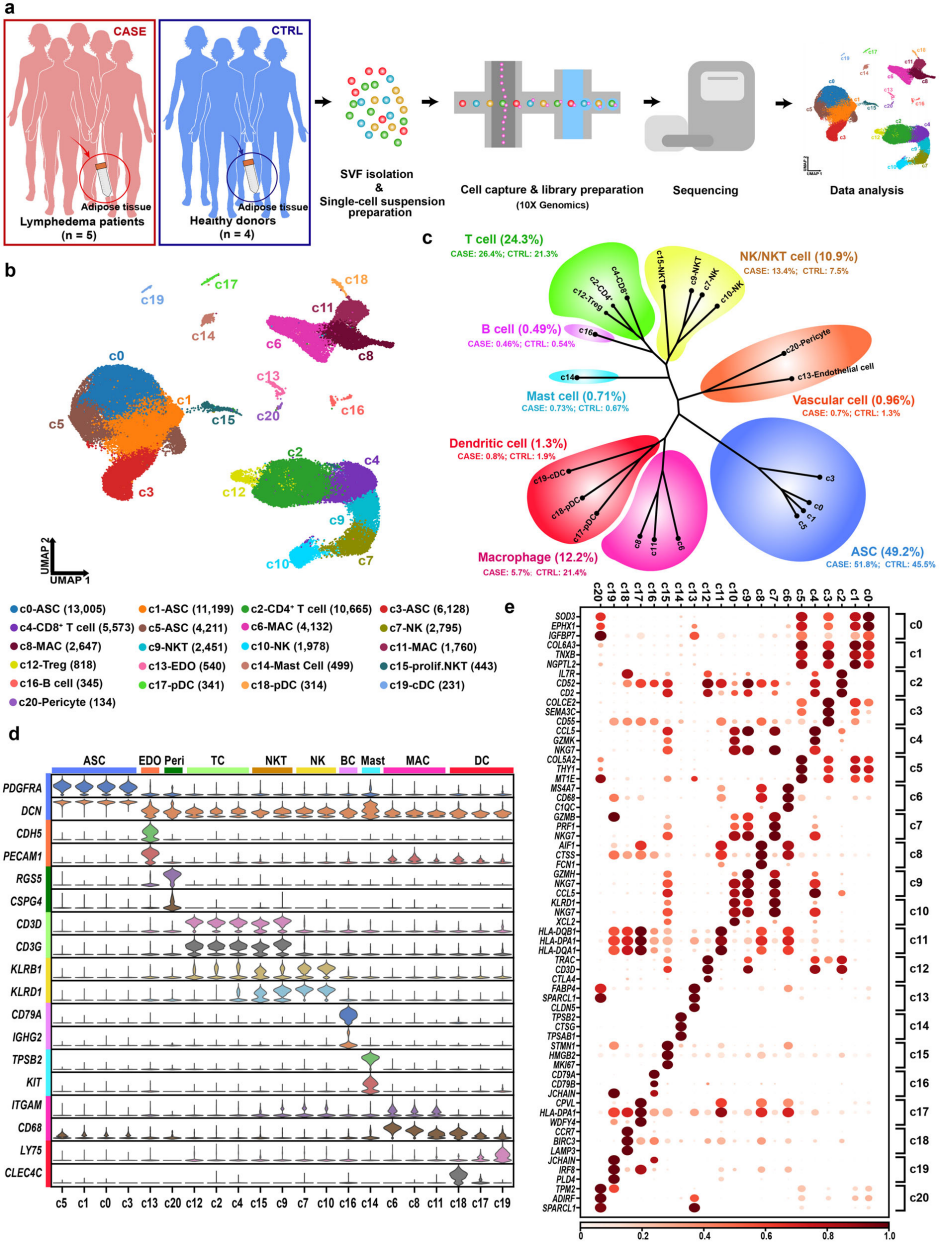
scRNA-seq reveals cellular diversity and heterogeneity of the SVF of adipose tissues in patients with cancer-related lymphedema. **a** Schematic representation of the experimental procedure. Patients with cancer-related lymphedema (*n* = 5; the CASE group) and healthy donors (*n* = 4; the CTRL group) were recruited in this study. Liposuction specimens from the thighs were collected during surgery. scRNA-seq was performed separately for each individual. **b** Unbiased clustering of 70,209 cells revealed 21 cellular clusters. The number in parentheses represents the cell count. **c** Hierarchical clustering of the clusters based on the mean expression of the 2000 most variable genes. **d** Expression of the established marker genes for each lineage in each cluster. **e** Representative molecular signatures for each cell cluster. The area of the circles indicates the proportion of cells expressing the gene, and the color intensity reflects the expression intensity. ASC, adipose-derived stromal/stem/progenitor cell; cDC, conventional dendritic cell; EDO, endothelial cell; MAC, macrophage; NK, natural killer cell; NKT, natural killer T cell; prolif.NKT, proliferative nature killer T cell; pDC, plasmacytoid dendritic cell.

To unbiasedly dissect cellular heterogeneity of the SVF of adipose tissues under healthy and diseased conditions, we obtained subcutaneous adipose tissue specimens from the affected thighs of 5 patients with severe lymphoedema (stage III; the CASE group) following surgical intervention for cervical cancer. As a control group (CTRL), liposuction specimens from the thighs of four healthy donors were also collected (Fig. 1a and Supplementary Table S1). There was no significant difference in BMI between the two groups (*P* value = 0.11, Wilcoxon rank-sum test). After SVF isolation, each fresh sample was subjected to scRNA-seq separately. Following stringent quality filtering, we ultimately obtained transcriptomes of 70,209 cells (CASE: 41,274 cells; CTRL: 28,935 cells). Unbiased clustering revealed 21 clusters (Fig. 1b). Based on hierarchical clustering (Fig. 1c) and established lineage-specific markers (Fig. 1d), we assigned these clusters to 10 cell lineages. The representative molecular signatures of these clusters are shown in Fig. 1e and Supplementary Table S2.

The ASC lineage (marked by *PDGFRA* and *DCN*)^19^, including c0, c1, c3, and c5, accounted for a large proportion (49.2%) of the SVF (Fig. 1c), which is comparable with that (55%) reported previously^6^. A large and diverse population of immune cells (49.9%) were found, including both myeloid cells and lymphocytes. The dominant lineage of myeloid cells was macrophages (marked by *ITGAM* and *CD68*)^20^, which included three subpopulations, i.e., c6, c8, and c11. Another two types of myeloid cells were mast cells (marked by *TPSB2* and *KIT*)^21^ and dendritic cells (DCs). The DCs encompassed clusters of conventional dendritic cells (c19; marked by *LY75*) and plasmacytoid dendritic cells (c18 and c17; marked by *CLEC4C*)^22^. The lymphocytes included T cells (c2, c4, and c12; marked by *CD3D* and *CD3G*)^23^, B cells (c16; marked by *CD79A* and *IGHG2*)^24^, natural killer (NK) cells (c7 and c10; marked by *KLRB1* and *KLRD1*)^25^ and natural killer T (NKT) cells (c9 and c15; expressing both NK and T cell markers). Detailed analysis revealed that both c2 and c12 belonged to CD4^+^ helper T cells (marked by *CD4* and *IL7R*; Supplementary Fig. S1). Cluster c12 also exhibited expression of *CTLR4* and *FOXP3* (Supplementary Fig. S1), thus representing a cluster of regulatory T cells (Treg cells)^26^. Cluster c4 was a cluster of CD8^+^ T cells, reflected by the high expression of *CD8A* and *CD8B* (Supplementary Fig. S1). The NKT cluster c15 expressed high levels of proliferation markers such as *MKI67* and *TOP2A*, thus representing proliferative NKT cells, whereas the NKT cluster c9 belonged to non-proliferative NKT cells (Supplementary Fig. S1). In addition, we identified vascular cells including endothelial cells (c13; marked by *CDH5* and *PECAM1*)^27^ and pericytes (c20; marked by *RGS5* and *CSPG4*)^28^. Since not enough lymphatic endothelial cells were identified, they could not be clustered separately.

### Differential proportional analysis reveals significantly expanded or contracted cell lineages and subpopulations in SVF associated with cancer-associated lymphedema

**Fig. 2:**
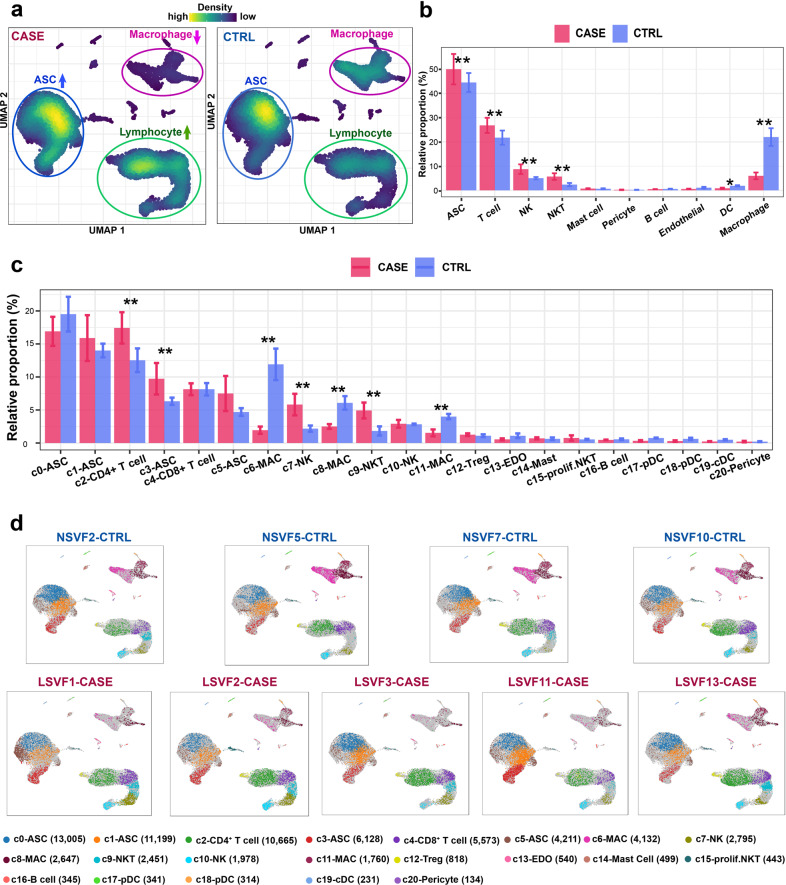
Differential proportional analysis reveals significantly expanded or contracted cell lineages and subpopulations associated with cancer-related lymphedema. **a** Visualization of the cellular density reveals dramatic changes in the proportions of multiple cell lineages in CASE versus CTRL. An equal number of cells (*n* = 28,935) were randomly sampled for each group. **b** Significantly expanded or contracted cell lineages. **c** Significantly expanded or contracted cellular clusters. **d** The distribution of cells for each cluster in each individual. In **b** and **c**, a permutation-based statistical test (differential proportion analysis) was performed. Data are represented as means ± SE. *Bonferroni-corrected *P* value < 0.05; **Bonferroni-corrected *P* value < 0.01. CASE, cancer-associated lymphedema; CTRL, healthy control.

Cell lineages that greatly change in relative proportion are probably associated with the pathogenesis of the disease. Visualization of the cellular density revealed dramatic changes in the relative proportions of multiple lineages in SVF, including ASCs, macrophages, and lymphocytes (Fig. 2a and Supplementary Fig. S2). To determine whether the change was expected by chance, we performed a permutation-based statistical test (differential proportion analysis) as described previously^29^. As shown in Fig. 2b, the ASCs were significantly expanded (Bonferroni-corrected *P* value < 0.01), which suggests enhanced proliferation of ASCs in lymphedema. Indeed, we observed significantly higher cycling scores for ASCs in CASE versus CTRL (*P* value = 4.916e−09, Wilcoxon rank-sum test; Supplementary Fig. S3). Strikingly, lymphocyte lineages (T cells, NK, and NKT cells) were significantly expanded, whereas the myeloid lineages (macrophages and DCs) were significantly contracted (Bonferroni-corrected *P* value < 0.05, differential proportion analysis; Fig. 2b). This result may reflect enhanced adaptive immunity at the advanced stage of lymphedema. Further analysis at the cluster level revealed significantly expanded subpopulations in SVF, including c2 CD4^+^ T cells; c3 ASCs, c7 NK cells, and c9 NKT cells, reflecting a strong association of these subpopulations with pathogenesis (Fig. 2c, d). The macrophage subpopulation cluster c6 was greatly contracted. Given the results above, we focused on the ASC and macrophage lineages, which may play critical roles in the pathogenesis, in the subsequent analysis.

### Heterogeneity of ASCs in the SVF of adipose tissues

**Fig. 3:**
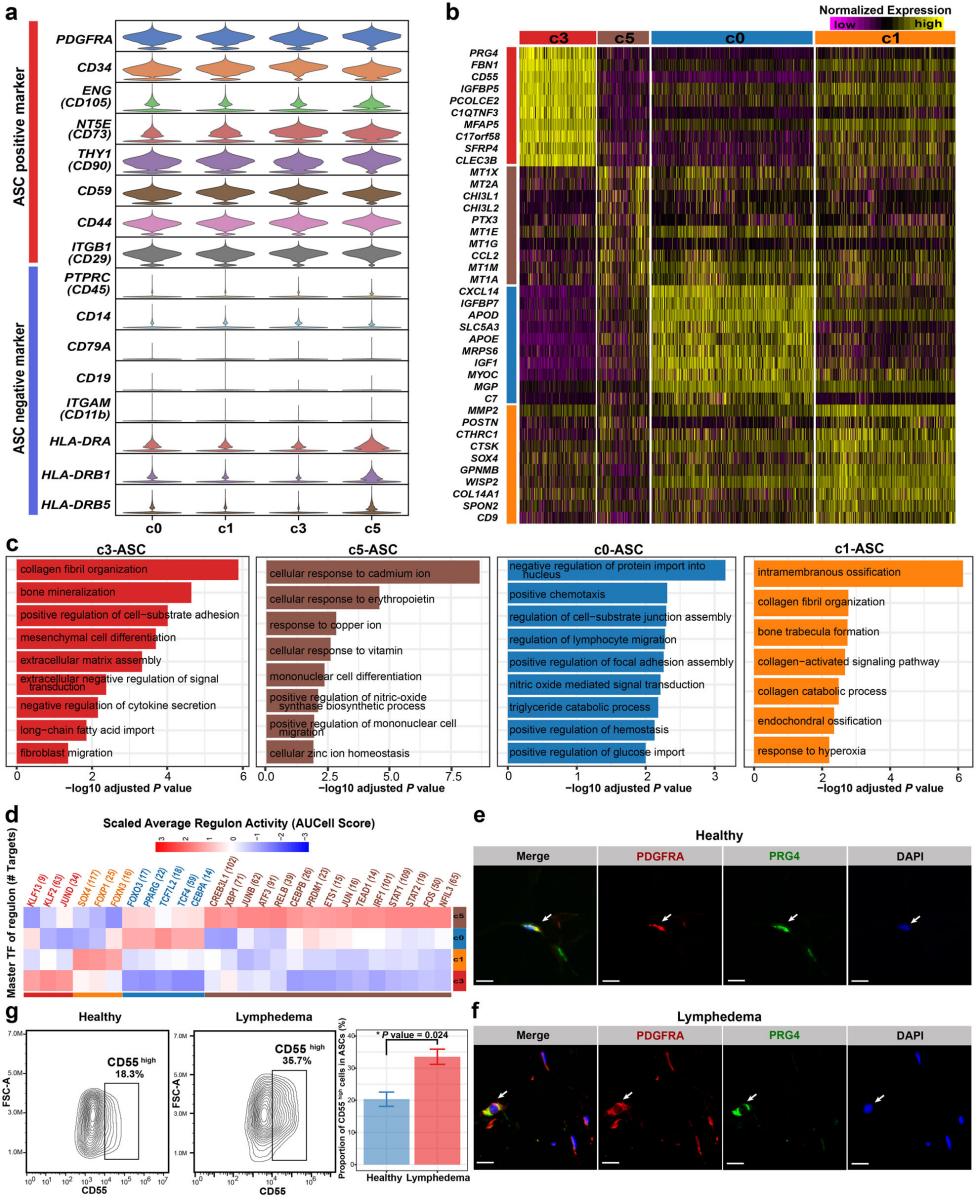
Heterogeneity of ASCs in adipose tissues revealed by single-cell analysis. **a** The expression of marker genes that are normally used for defining freshly isolated or cultured ASCs. **b** Distinct expression profiles of the four subpopulations of ASCs. **c** Enriched Gene Ontology terms of the molecular signature for each subpopulation. Adjusted *P* value < 0.05, the hypergeometric test. **d** Subpopulation-specific regulons of each subpopulation obtained by SCENIC analysis. **e** Immunofluorescence staining confirmed the presence of the c3-ASC subpopulation in the subcutaneous adipose tissues of lymphedema patients. Staining was performed in tissue sections of three patients. **f** Immunofluorescence staining confirmed the presence of the c3-ASC subpopulation in the subcutaneous adipose tissues of healthy donors. Staining was performed in tissue sections of two healthy donors. In **e**, **f**, one representative staining image of one subject is shown. Arrows indicate the *PDGFRA*^+^*PRG4* ^high^ cells. Scale bar: 20 μm. **g** Flow cytometry analyses of the expression of CD55 (a marker for c3-ASC) in ASCs under lymphedema and healthy conditions. A validation cohort (Validation cohort I; *n* = 4 for the lymphedema group and *n* = 7 for the healthy group) independent of the samples subjected to scRNA-seq were used in this analysis. The left two panels show representative contour plots for each group. The bar plot indicates a significantly higher proportion of CD55^high^ cells in ASCs (PDGFRα^+^) from lymphedema patients than that from healthy donors. Data are presented as means ± SE. *P value < 0.05. Wilcoxon rank-sum test. FSC-A forward scatter-area.

We examined the expression of established markers for freshly isolated or cultured ASCs (Fig. 3a). Consistent with our knowledge, *CD34*, a marker for freshly isolated ASCs^30^, is highly expressed in all ASC subpopulations, suggesting that our isolated cells were close to the in vivo state. The ASCs expressed positive markers for the definition of cultured ASCs (e.g., *CD105*, *CD73*, *CD90*, *CD59*, *CD44*, and *CD29*) and generally lacked expression of negative markers (e.g., *CD45*, *CD14*, *CD11b*, *CD19*, and *CD79A*)^31,32^. Notably, we found that some ASCs, particularly in cluster c5, expressed MHC class II genes (e.g., *HLA-DRA*, *HLA-DRB1*, and *HLA-DRB5*), suggesting that these cells had antigen-presenting functions. This finding agrees with the notion that antigen-presenting could be induced under inflammation or disease conditions for ASCs^33^. Next, we found that the four subpopulations of ASCs had distinct expression profiles (Fig. 3b and Supplementary Table S3). Cluster c0 expressed high levels of preadipocyte markers such as *CXCL14*, *APOD*, *APOE*, *MGP*, and *WISP2*^6^. The gene signature of c0 was enriched with the term “triglyceride catabolic process” (Fig. 3c). Regulon analysis using SCENIC^34^ identified *PPARG* and *CEBPA*, the known master TFs in adipogenesis^35^, as c0-specific regulators (Fig. 3d and Supplementary Table S4). *GGT5*, the maker for committed preadipocytes reported previously^36^, was highly expressed in c0 (Supplementary Fig. S4). As such, c0 represents committed preadipocytes. Notably, c3, a lymphedema-associated ASC subpopulation that was significantly expanded in SVF (Fig. 2c) and the ASC lineage (Supplementary Fig. S5a), showed high expression of *DPP4* and *CD55*, two markers for multipotent interstitial progenitor cells in adipose tissues^36^ (Supplementary Fig. S4). It also expressed *PRG4*, which encodes a secreted mucinous glycoprotein^37^. Its molecular signature was enriched with GO terms such as “collagen fibril organization”, “bone mineralization” and “mesenchymal cell differentiation” (Fig. 3c). As such, c3 may represent interstitial progenitor cells closely associated with the fibrosis of adipose tissues in lymphedema. Through immunofluorescence staining, we confirmed the presence of the *PRG4*^+^ ASCs in subcutaneous adipose tissues of healthy donors (Fig. 3e and Supplementary Fig. S6) and lymphedema patients (Fig. 3f and Supplementary Fig. S6). Based on the spatial distribution in the uniform manifold approximation and projection (UMAP) embedding and the expression of markers (Supplementary Fig. S4), c1 may represent intermediate states between interstitial progenitor and committed preadipocytes. Cluster c5 was an ASC subpopulation displaying a unique pattern with a high expression of metallothionein genes such as *MT1X*, *MT2A*, *MT1E*, *MT1G*, *MT1M*, and *MT1A* (Fig. 3b). Given that metallothionein proteins mainly play roles in protection against damage associated with heavy metal toxicity, endoplasmic reticulum stress, or oxidative stress^38,39^, c5 may represent a stress-responsive subpopulation. The presence of c1, c3, and c5 cells was also confirmed by immunofluorescence staining in the subcutaneous adipose tissue sections from lymphedema patients and healthy donors (Supplementary Figs. S7–S9). In addition, the expression of the markers of the four ASC subpopulations could be observed in cellular subclusters of a published single-cell dataset of human subcutaneous adipose tissue (Supplementary Fig. S10), thus our results represent true heterogeneity in ASCs with improved resolution compared with the previous report (only two subgroups)^36^. In addition, in a validation cohort (validation cohort I; *n* = 4 for the lymphedema group; *n* = 7 for the healthy group; Age and BMI were matched between groups, *P* value > 0.05, Wilcox rank-sum test; Supplementary Table S5), through flow cytometry analyses, we confirmed that there was a significantly higher proportion of CD55^high^ cells (c3-ASCs) in ASCs (PDGFRα^+^) from lymphedema patients than that from healthy donors (Fig. 3g and Supplementary Fig. S11; *P* value < 0.05, Wilcox rank-sum test).

### Dysregulated pathways and genes in the ASCs of cancer-associated lymphedema

**Fig. 4:**
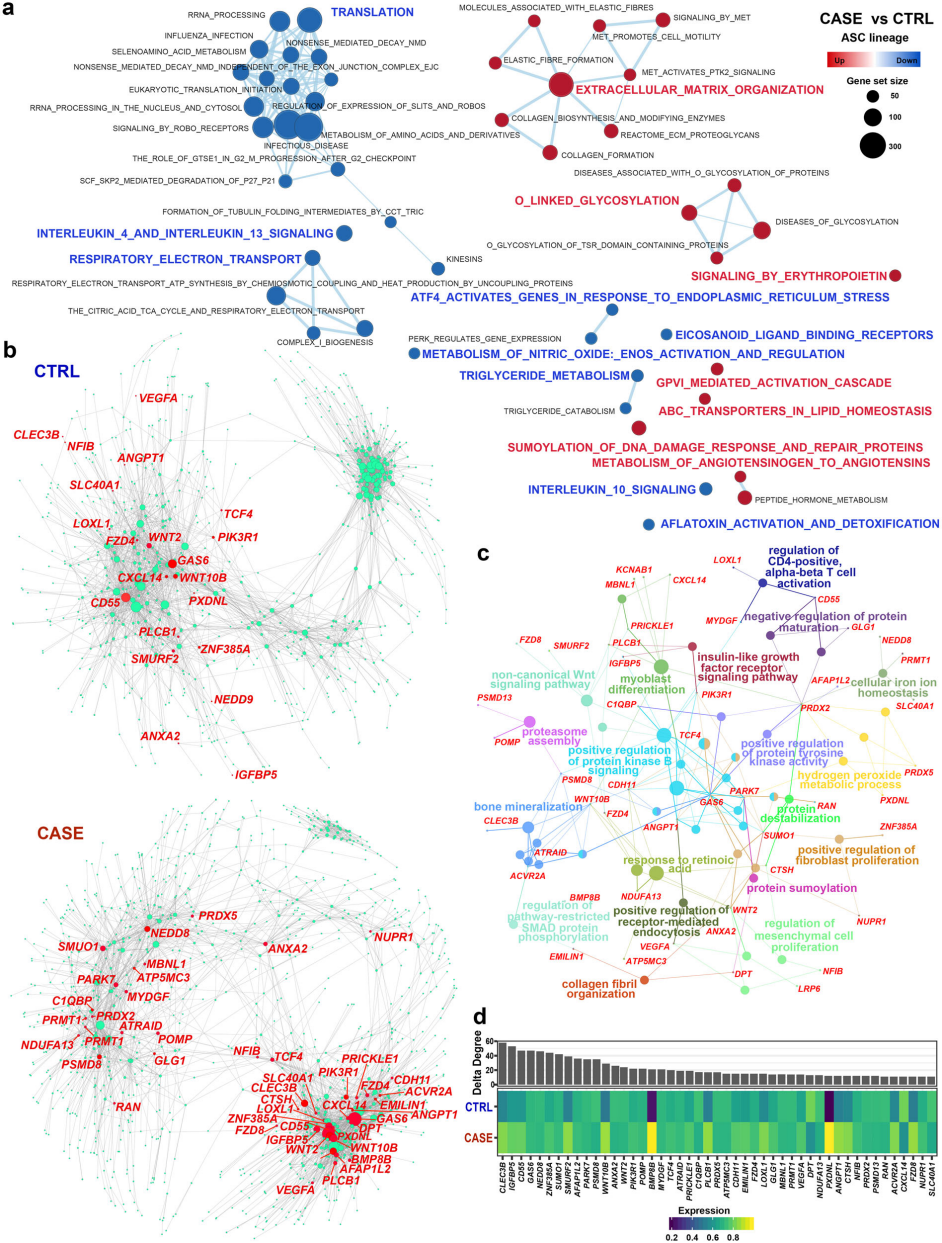
Dysregulated genes and pathways of ASCs in adipose tissues from cancer-related lymphedema. **a** GSEA reveals upregulated or downregulated pathways of ASCs in CASE versus CTRL. An FDR *q* value < 0.05 was considered to be statistically significant. **b** Comparative analysis of the gene regulatory networks of ASCs between the CASE (lower panel) and CTRL (upper panel) groups reveals dysregulated genes in ASCs. The node size reflects the degree centrality. The representative genes dysregulated in CASE ranked by delta degree are labeled in red. **c** Network view of the functional enrichment for the dysregulated genes shown in **b**. The small dots denote genes and large nodes represent Gene Ontology terms. The node size represents the number of genes associated with the Gene Ontology term. Adjusted *P* value < 0.05. **d** Delta degree centrality (upper panel) and average expression across cells in CASE and CTRL (lower panel). CASE, cancer-associated lymphedema; CTRL, healthy control.

scRNA-seq allows unbiased analysis of lineage-specific transcriptomic changes in diseased conditions without cell sorting. We next explored the dysregulated pathways through gene set enrichment analysis (GSEA), which facilitates biological interpretation by robustly detecting concordant differences at the pathway level^40^. Extracellular matrix-related pathways such as “extracellular matrix organization” and “collagen formation” were significantly upregulated (FDR *q* value < 0.05, GSEA; Fig. 4a and Supplementary Table S6), which is in line with the fibrosis of adipose tissues in lymphedema. Glycosylation is a common modification of proteins and lipids, which has been implicated in physiological (e.g., cell differentiation) and pathophysiological states (e.g., autoimmunity and chronic inflammation)^41^. Strikingly, glycosylation-related pathways such as “*O*-linked glycosylation” and “diseases of glycosylation” were significantly upregulated, which suggests that increased glycosylation or altered glycosylation patterns in ASCs may contribute to pathogenesis. In addition, “SUMOylation of DNA damage response and repair proteins” was upregulated, reflecting the DNA damage induced by chronic inflammation^42^. Compared with the healthy condition, ASCs in lymphedema displayed downregulated protein translation, energy metabolism, and response to endoplasmic reticulum stress (Fig. 4a), reflecting impaired cellular functions at the advanced stage of lymphedema. Unexpectedly, we found decreased adipogenesis for ASCs in lymphedema, as evidenced by the significantly decreased expression of *PPARG* and *CEBPA* (Supplementary Fig. S12a, b), the master regulators in adipogenesis^10^ as well as significantly decreased adipogenesis score (*P* value < 2.2e−16, Wilcoxon rank-sum test; Supplementary Fig. S12c). In addition, we found significantly increased osteogenesis of ASCs in lymphedema (Supplementary Fig. S12d), which reflects aberrant differentiation in diseased conditions.

Next, we built gene regulatory networks from the single-cell data using a novel method implemented in bigScale2^43^ which allows us to quantify the biological importance of genes and find dysregulated genes in diseased conditions. The regulatory networks constructed for ASCs in healthy (upper panel) and diseased conditions (lower panel) are shown in Fig. 4b. Comparative analysis between the two networks revealed a list of genes that were greatly increased in degree centrality (the number of edges connected to a given node; Fig. 4b and Supplementary Table S7) in lymphedema, reflecting their potential roles in the pathogenesis. These genes were mainly involved in bone mineralization, positive regulation of protein kinase B signaling, and regulation of mesenchymal cell proliferation and differentiation (Fig. 4c). Notably, C-Type Lectin Domain Family 3 Member B (*CLEC3B*) ranked at the top of the list based on the changes in degree centrality (Fig. 4d). The expression of *CLEC3B* was upregulated in CASE compared to CTRL (Fig. 4d) and was especially high in the lymphedema-associated subpopulation c3 (Fig. 3b and Supplementary Table S3), which implies that *CLEC3B* may be associated with fibrosis. The elevated expression of *CLEC3B* was confirmed by using a “pseudobulk” method^44^ (“edgeR-lrt”) implemented in edgeR that accounts for variations among biological replicates (Supplementary Fig. S13a; *P* value = 0.05). In a validation cohort (validation cohort II, Supplementary Table S5), the expression of *CLEC3B* was significantly upregulated in ASCs freshly isolated from the adipose tissues (passage 0-ASCs) of the lymphedema group (*n* = 3) versus the healthy group (*n* = 3) (Supplementary Fig. S13b; *q* value = 0.026, differential expression test). In addition, significantly upregulated expression of *CLEC3B* was observed in cultured ASCs (passage 3) from the lymphedema versus normal adipose tissues of patients with secondary lymphedema (*n* = 8) based on the bulk RNA-seq data previously generated by our lab^45^ (Supplementary Fig. S13c; *P* value = 0.024, Wilcoxon signed-rank test). These evidences support our hypothesis that *CLEC3B* may be a candidate target for lymphedema.

### Knockdown of CLEC3B could significantly attenuate the fibrogenesis of ASCs from patients

**Fig. 5:**
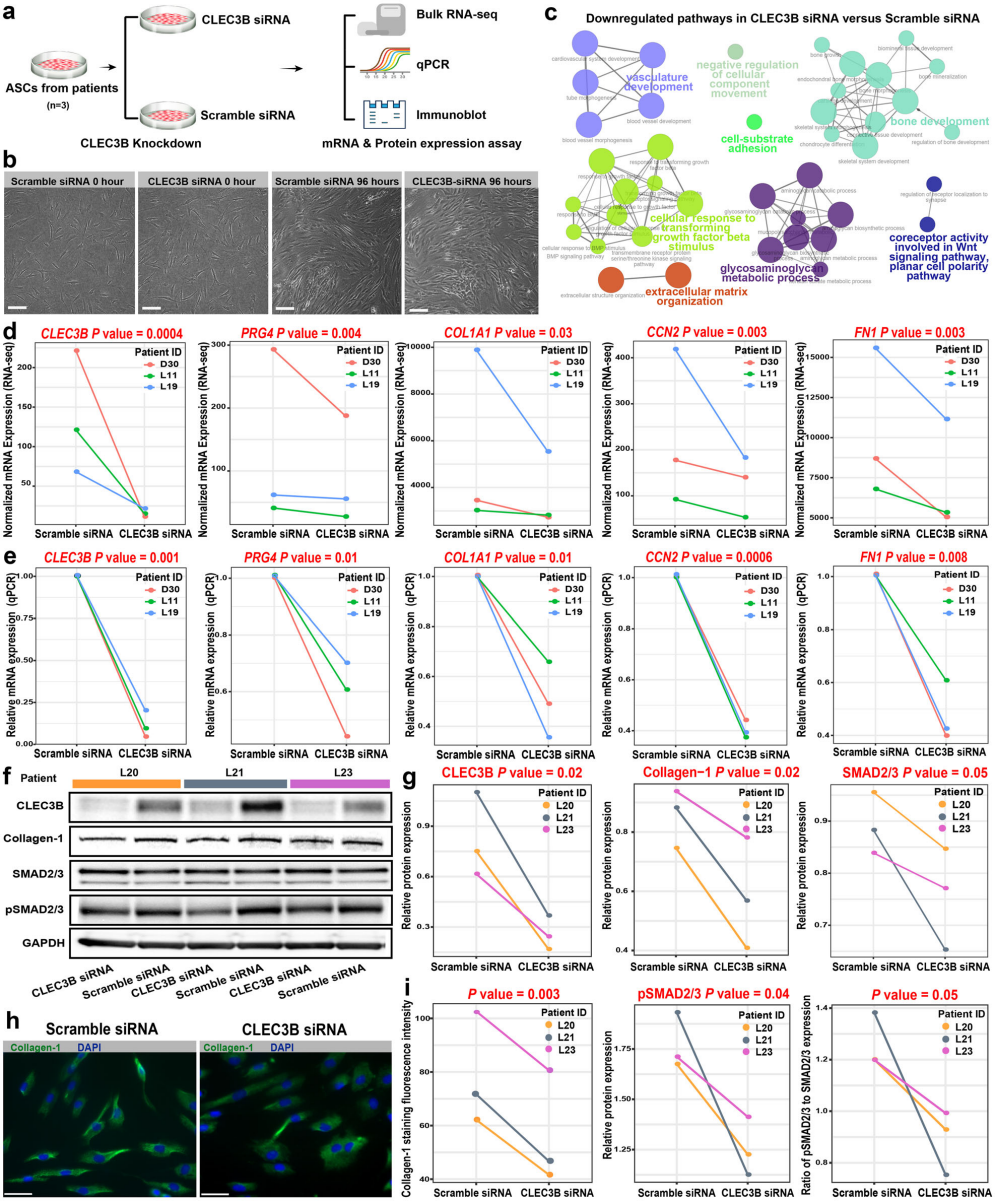
Knockdown of *CLEC3B* could significantly attenuate the fibrogenesis of ASCs from lymphedema patients. **a** Schematic representation of the experimental procedure. ASCs from patients (*n* = 3) were transfected with CLEC3B siRNA or scramble siRNA (negative control). The cells were harvested 48 h and 96 h after transfection for mRNA and protein analysis, respectively. **b** Images showing the ASCs after 96 h of transfection. Scale bar: 100 μm. **c** Network plot showing the downregulated pathways in ASCs with siRNA-mediated knockdown of *CLEC3B* versus the negative control. Functional enrichment analysis was performed on the differential expressed genes detected by bulk RNA-seq. Adjusted *P* value < 0.05, the hypergeometric test. **d** Significantly reduced mRNA expression of c3 markers and fibrosis-related genes in ASCs with *CLEC3B* knockdown versus the negative control (determined by bulk RNA-seq). The significance threshold was set to be a *P* value < 0.05 of the sleuth differential expression test. **e** Significantly reduced mRNA expression of c3 markers and fibrosis-related genes in ASCs with *CLEC3B* knockdown versus the negative control (determined by qPCR). The significance threshold was set to be a *P* value < 0.05 of the one-tailed paired *t*-test. **f** Immunoblot assays showing the decreased protein expression of CLEC3B, Collagen-1, SMAD2/3, and phosphorylated SMAD2/3 in ASCs with *CLEC3B* knockdown versus the negative control (*n* = 3). **g** Densitometry analysis of the immunoblot results. The significance threshold was set to be a *P* value < 0.05 of the one-tailed paired *t*-test. **h** Immunofluorescent staining of Collagen-1 in ASCs transfected with CLEC3B siRNA or scramble siRNA. Scale bar: 50 μm. Representative fields of view of ASCs from the patient L21 are shown. **i** Collagen-1 staining fluorescence intensity comparison. Each value represents the mean fluorescence intensity of three representative fields of view. Two-tailed paired *t*-test.

To test the hypothesis that *CLEC3B* is associated with fibrosis, we performed a siRNA-mediated knockdown of *CLEC3B* in ASCs isolated from lymphedema patients (Fig. 5a). The ASCs were in a good state after 96 h of transfection (Fig. 5b). Through bulk RNA-seq, we identified 488 differentially expressed genes between ASCs with the knockdown of *CLEC3B* and the negative control (*q* value < 0.05, differential expression test; Supplementary Table S8). The extracellular matrix-related pathways (e.g., “extracellular matrix organization”) were significantly downregulated (adjusted *P* value < 0.05, the hypergeometric test; Fig. 5c), suggesting that the fibrogenesis of ASCs was attenuated by the knockdown of *CLEC3B*. Furthermore, we examined the mRNA expression changes of some representative genes determined by bulk RNA-seq (Fig. 5d). Intriguingly, concomitant with the knockdown of *CLEC3B*, the mRNA expression of *PRG4* (one marker of c3 subpopulation) was also significantly downregulated (*P* value < 0.05, differential expression test). The expression of the established fibrosis-related genes such as *COL1A1*, *CCN2*, and *FN1* was significantly downregulated. The mRNA expression changes of these genes were confirmed by quantitative real-time polymerase chain reaction (qPCR; Fig. 5e, *P* value < 0.05, paired *t*-test). In addition, we examined the protein expression changes using immunoblot (Fig. 5f, g). The protein level of CLEC3B was significantly decreased by knockdown. The protein levels of Collagen-1 (a fibrosis effector protein), SMAD2/3, and phosphorylated SMAD2/3 (pSMAD2/3, the key regulators in the canonical TGFβ pathway of fibrosis) were significantly decreased (*P* value < 0.05, paired *t*-test). The mean fluorescence intensity of Collagen-1 staining in ASCs transfected with CLEC3B siRNA significantly decreased (Fig. 5h, i, *P* value < 0.05, paired *t*-test). Together, our results suggest that *CLEC3B* could serve as a potential target for alleviating the fibrosis of adipose tissues in lymphedema.

### Adipose tissues of lymphedema display a striking depletion of anti-inflammatory macrophages and exhibit a pro-inflammatory microenvironment

We next explored the phenotypic differences among the three lymphedema-associated macrophage subpopulations (c6, c8, and c11). These subpopulations displayed distinct expression profiles (Supplementary Fig. S14a and Table S9). Compared with other subpopulations, c6 showed high expression of *LYVE1*, a marker gene for tissue-resident macrophages^46^. It also displayed a high expression of markers for M2-polarized (alternatively activated) macrophages, including *RNASE1*, *SELENOP*, *MRC1*, and *CD163* (Supplementary Fig. S14b), which harbor an anti-inflammatory phenotype^47^. Thus, the *LYVE1*^+^ c6 cluster represented a resident-like macrophage subpopulation with an M2 phenotype. Compared with the others, cluster c8 expressed higher levels of *IL1B*, a pro-inflammatory cytokine, and markers for M1-polarized (classically activated) macrophages such as *FCGR1A*, *TNF*, and *FPR2*^48^. The *IL1B*^high^ cluster c8 thus represented a pro-inflammatory macrophage subpopulation with an M1 phenotype. Cluster c11 expressed high levels of *CD1C*, encoding an antigen-presenting molecule, and MHC class II genes (e.g., *HLA-DQA1*, *HLA-DPB1*, and *HLA-DPA1*; Supplementary Fig. S14a). It expressed both M1 and M2 markers, e.g., *CD86* and *MRC1*, respectively (Supplementary Fig. S14b). The molecular signature of c11 was enriched with antigen presentation-related terms such as “antigen processing and presentation of exogenous antigen” (Supplementary Fig. S14c). These results suggest that the *CD1C*^high^ cluster c11 represented a specialized antigen-presenting macrophage subpopulation. Furthermore, we identified subpopulation-specific regulons through SCENIC analysis (Supplementary Fig. S14d and Table S10), for example, *CEBPB*, *FOSL2*, *STAT1*, and *IRF7* for the pro-inflammatory macrophage subpopulation c8. As mentioned above, the macrophage subpopulation c6 was dramatically reduced in SVF (Fig. 2c) and the macrophage lineage under lymphedema (Supplementary Fig. S5b). We calculated the ratio of c6/c8, as a proxy of the ratio of M2/M1, and found that it was greatly decreased in lymphedema (0.76 in CASE versus 2.03 in CTRL), reflecting a pro-inflammatory microenvironment in lymphedema.

### Pharmacological blockage of Trem1 using mLR12 could significantly alleviate the lymphedema in a mouse tail model

**Fig. 6:**
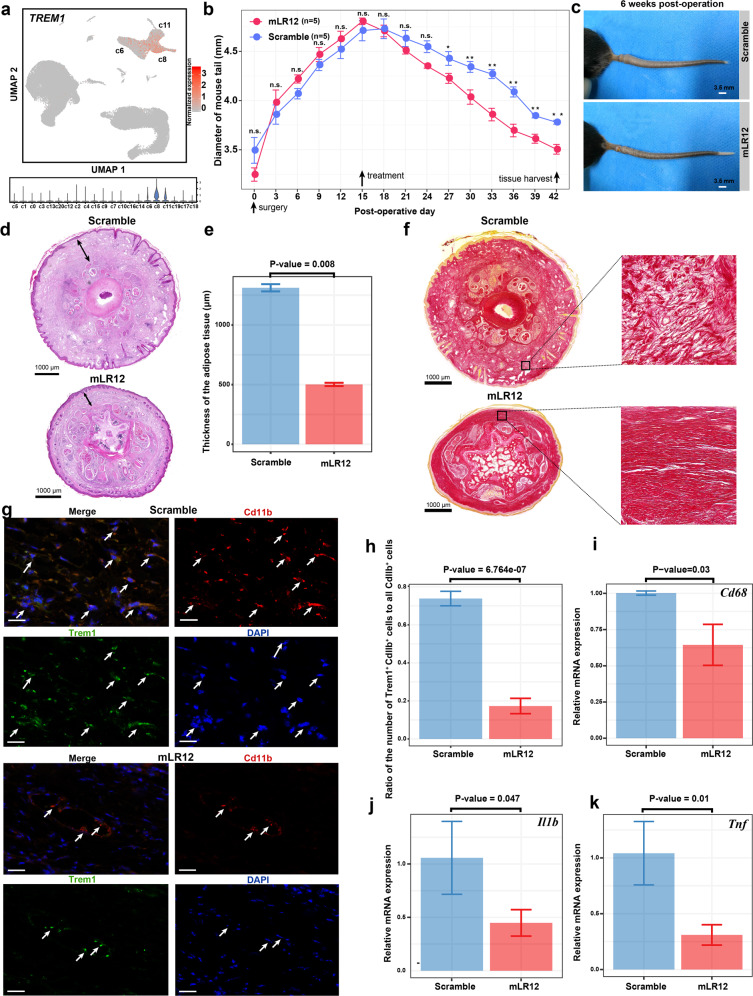
Pharmacological blockage of Trem1 using mLR12 could significantly alleviate the lymphedema in a mouse tail model. **a** *TREM1* is predominantly expressed by the pro-inflammatory macrophage subpopulation c8. **b** Changes in the diameter of mouse tail during the treatment using mLR12 (*n* = 5) or mLR12-scramble peptides as a control (*n* = 5). Starting from 15 days after the surgery, mice were treated daily by intraperitoneal injection. The diameter of the tail (1 cm distal to the incision) was measured every 3 days for 6 weeks. Tail tissue samples were collected 6 weeks after the surgery for subsequent assays. *P value < 0.05, **P value < 0.01, n.s. not significant, one-tailed *t*-test. **c** Images showing the mouse tails following treatment. **d** H&E staining of the cross-sections of the mouse tails showing reduced subcutaneous tissue edema in the treatment versus the control group. The double-sided arrow indicates the thickness of subcutaneous tissues. **e** Thickness of the subcutaneous tissues measured based on the H&E staining images. **f** Sirius red staining showing decreased fibrosis. Collagens were stained in red. Collagen fibers are disarrayed in the fibrosis of the scramble group. **g** Immunofluorescent staining showing a reduced number of Cd11b^+^Trem1^+^ cells in the treatment group (lower panel) versus the control group (upper panel). The arrows indicate the Cd11b^+^Trem1^+^ cells. **h** Proportion of the Cd11b^+^Trem1^+^ macrophages in all Cd11b^+^ cells in the two groups based on the immunofluorescent staining. **i** Reduced mRNA expression of *Cd68* in the mouse tail tissue following the LR12 treatment. **j** Reduced mRNA expression of *Il1b* in the mouse tail tissue following the LR12 treatment. **k** Reduced mRNA expression of *Tnf* in the mouse tail tissue following the LR12 treatment. In **e**, **h**–**k**, the significant threshold was set to be a *P* value of the one-tailed *t*-test < 0.05 (*n* = 3). In **d**, **e**, sections at almost the same level (located 1 cm distal to the surgical site) were used for both groups.

Given the pro-inflammatory microenvironment of adipose tissues in lymphedema, we next explored the effects of therapies targeting the immune cells. We noted that the pro-inflammatory macrophage subpopulation c8 predominantly expressed *TREM1* (Fig. 6a), which encodes an immune receptor that amplifies the inflammatory response and regulates myeloid recruitment into the inflammatory site^49^. LR12 peptide, a decoy receptor that effectively blocks TREM1 engagement, has recently been proved to be effective in treating sterile inflammation-related diseases such as atherosclerosis^50^. We thus evaluated the effects of pharmacological blockade of Trem1 using murine LR12 (mLR12) in a widely used mouse tail model of lymphedema. Strikingly, the mouse tail diameter (an indicator of the edema degree) curve of the mLR12 treatment group gradually separated from that of the scramble group following treatment (Fig. 6b). Treated with mLR12, the lymphedema in the mouse tail was greatly alleviated in 6 weeks after the surgery (Fig. 6c). H&E staining of the cross-sections of the mouse tails showed significantly reduced subcutaneous tissue thickness in the treatment versus the control group (Fig. 6d, e). While collagen fibers were disarrayed in the fibrosis of the scramble group, Sirius red staining showed decreased fibrosis in the treatment group (Fig. 6f). Immunofluorescent staining confirmed the reduced number of Cd11b^+^Trem1^+^ cells in the mouse tail tissue of the treatment group versus the control (Fig. 6g, h). The expression of *Cd68* in the mouse tail tissue of the treatment group was significantly decreased compared with that of the scramble group, indicating reduced infiltration of macrophages and inflammation. In addition, the expression of the genes encoding detrimental cytokines such as *Il1b* and *Tnf* was also significantly decreased with the treatment of mLR12 (Fig. 6j, k). Together, these results suggested that the pharmacological blockage of Trem1 using mLR12 could significantly reduce inflammation and fibrosis, thereby alleviating the lymphedema in mice.

### Cell–cell communication analysis uncovers a perivascular ligand-receptor interaction module and communication changes for ASCs in cancer–associated lymphedema

**Fig. 7:**
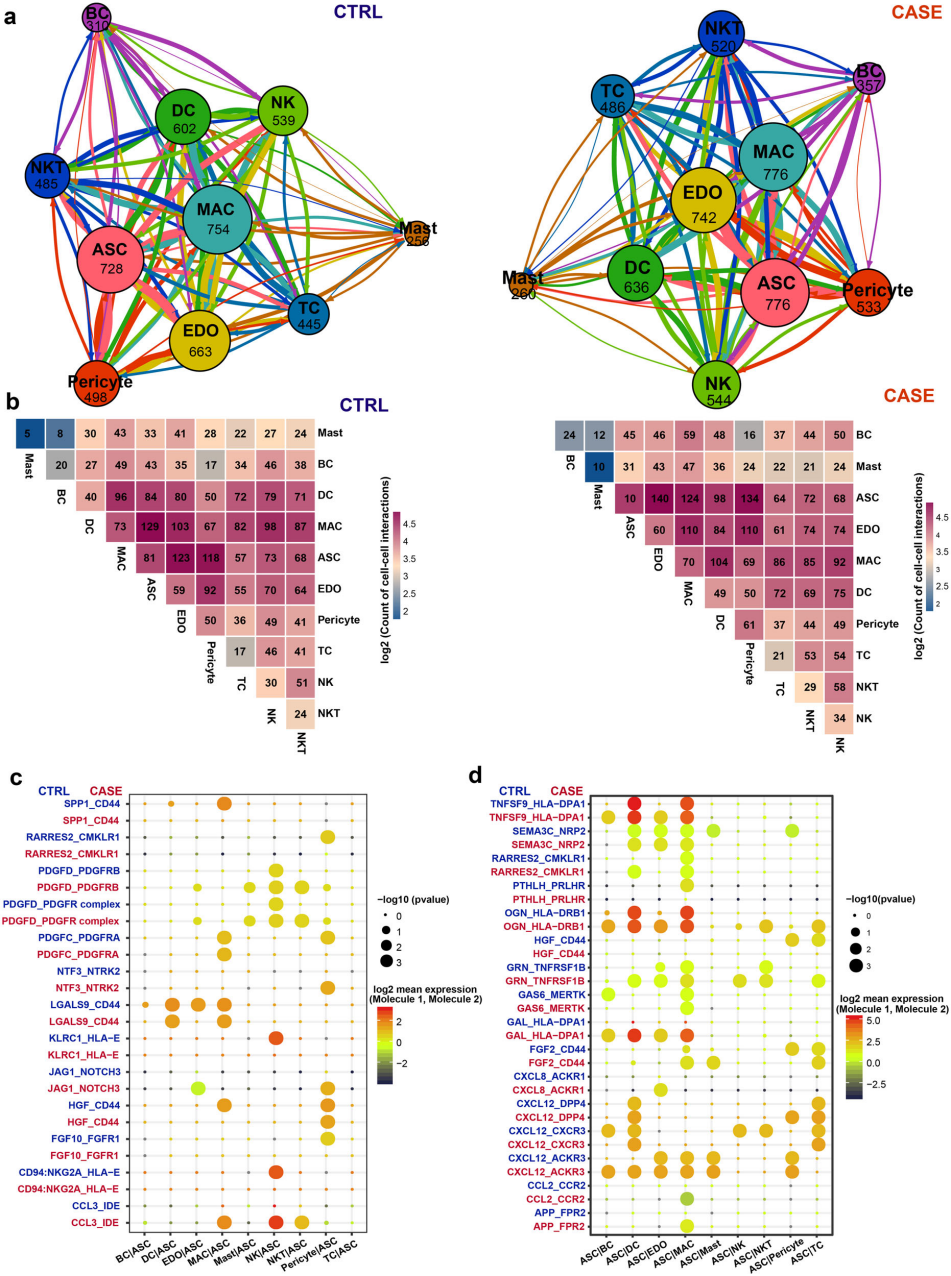
Cell–cell communication analysis reveals a perivascular ligand–receptor interaction module and communication changes for ASCs in cancer-related lymphedema. **a** Intercellular communication networks in the adipose tissues from patients with lymphedema (CASE; right panel) and healthy people (CTRL; left panel). The total number of communications is shown for each cell lineage. The line color indicates that the ligands are broadcast by the cell lineage in the same color. The line thickness is proportional to the number of broadcast ligands. **b** Heatmap shows the number of communications between any two lineages in the CASE (right panel) and CTRL (left panel) groups. **c** The ligand–receptor pairs showing significant changes in specificity between any one of the non-ASC lineages and ASCs in CASE versus CTRL. ASCs express receptors and receive ligand signals from other lineages. The dot size reflects the *p* value of the permutation tests for lineage specificity. The dot color denotes the mean of the average ligand–receptor expression in the interacting lineages. **d** The ligand–receptor pairs showing significant changes in specificity between ASCs and any one of the non-ASC lineages in CASE versus CTRL. ASCs express ligands and broadcast ligand signals for other lineages. ASC, adipose-derived stromal/stem/progenitor cell; BC, B cell; CASE, cancer-associated lymphedema; CTRL, healthy control; DC, dendritic cell; EDO, endothelial cell; MAC, macrophage; NK, natural killer cell; NKT, natural killer T cell; TC, T cell.

To define the cell–cell communication landscape and uncover its alterations in lymphedema, we performed an analysis using CellPhoneDB 2.0^51^. Strikingly, we identified a densely connected communication network among macrophages, ASCs, and vascular endothelial cells in both conditions (Fig. 7a), which is concordant with our knowledge that macrophages, especially *LYVE1*^+^ macrophages^46^, and ASCs^52^ are spatially associated with the blood vasculature. In line with this, we found that ASCs were the predominant source of the macrophage colony-stimulating factor CSF1 (Supplementary Fig. S15a), which is critical for the survival of macrophages^53^. The expression of *CSF1* in ASCs was significantly higher in lymphedema than in the healthy condition (Supplementary Fig. S15b), reflecting enhanced signals broadcast by ASCs under the diseased condition. We, therefore, identified a perivascular ligand–receptor signal module. Compared with the healthy condition, the total number of interactions for almost all lineages increased in lymphedema (Fig. 7a), reflecting enhanced intercellular communications in diseased conditions. Notably, the most abundant interactions in the network occurred between ASCs and macrophages under the healthy condition, whereas the most abundant interactions occurred between ASCs and vascular endothelial cells in lymphedema (Fig. 7b). Furthermore, we identified the ligand–receptor pairs showing significant changes in specificity between any one of the non-ASC lineages and ASCs in diseased versus healthy conditions (ASCs express receptors and receive ligand signals from other lineages; Fig. 7c and Supplementary Table S11). Notably, PDGFD–PDGFR complex interactions were significantly enhanced between some lineages (vascular endothelial cells, mast cells, NKT cells, and pericytes) and ASCs in lymphedema. Increased secretion of PDGFD or enhanced PDGFD signaling has been associated with aberrant proliferation and differentiation of fibroblasts in some diseases such as fibrosis and cancer^54,55^. Our results suggest that PDGFD signaling may contribute to the enhanced fibrosis and proliferation of ASCs in lymphedema. In addition, we also explored the alterations in ligand signals broadcast by ASCs (Fig. 7d). Notably, some chemokine signals broadcast by ASCs, including CXCL8, CXCL12, and CCL2, were significantly altered. For example, CXCL12–ACKR3 interactions between ASCs and BCs or DCs become significantly more specific in lymphedema (permutation test *P* value < 0.05).

## Discussion

Lymphedema is characterized by excess adipose deposition in the affected limbs^8^; however, the underlying mechanism remains elusive. Previous studies suggested enhanced adipogenesis, i.e., the differentiation of adipocytes from ASCs in mouse models^12^ and human patients^10^, based on a limited number of marker genes. In contrast, our single-cell analysis did not find any significantly upregulated pathways associated with adipogenesis. Instead, we found that ASCs from lymphedema may have decreased adipogenesis (Supplementary Fig. S12) and enhanced proliferation ability (Supplementary Fig. S3). The enhanced proliferation of ASCs from lymphedema is consistent with the finding of a study based on bulk RNA-seq^45^. Histological evidence has shown that hypertrophic (cell enlargement) adipocytes are frequently observed, especially in the severe stages of lymphoedema^9^. Therefore, we think that the excess adipose deposition may be mostly attributed to the enhanced proliferation ability of ASCs and cell enlargement of adipocytes at least in the severe stage of lymphoedema.

Stage III lymphedema, also known as lymphostatic elephantiasis, is a severe condition in which the tissue becomes extremely swollen, thickened, and fibrotic^1^. Concordant with the enhanced fibrosis, we found that the extracellular matrix-related pathways, such as “extracellular matrix organization” and “collagen formation”, were significantly upregulated in ASCs from lymphedema (Fig. 4a). Furthermore, we pinpointed the ASC subpopulation that was closely associated with the pathophysiology of lymphedema, i.e., c3 (*PRG4*^+/^*CLEC3B*^+^ ASCs), which was significantly expanded in lymphedema. It showed high expression of *DPP4* and *CD55*, two markers for multipotent interstitial progenitor cells in adipose tissue^36^ (Supplementary Fig. S4b), suggesting that it generally corresponds to multipotent interstitial progenitors. We also noted that the molecular signature of this subpopulation was enriched with pathways such as “collagen fibril organization” and “bone mineralization” (Fig. 3c), suggesting that this subpopulation was related to fibrosis and pathologic mineralization of adipose tissues in lymphedema. However, it does not correspond to fibro-inflammatory progenitors (FIPs) previously identified in visceral white adipose tissue of adult mice^56^, since it expressed high levels of some FIP markers, for example, fibrosis-associated markers, *FN1* and *LOXL1*, but exhibited low expression for other markers, for example, inflammation-associated marker *CCL2* (Supplementary Fig. S4c). It has been suggested that the multipotent interstitial progenitors of visceral white adipose tissues were depleted by obesity and/or high-fat feeding^36^. Our study reported the expansion and dysregulation of the multipotent interstitial progenitors of subcutaneous adipose tissues in response to the pathophysiological stimuli of lymphedema. The balance between multipotent interstitial progenitors and preadipocytes is therefore essential for the homeostasis of adipose tissues. The development of medical therapies to balance the two groups of cells may be a novel therapeutic strategy. We demonstrated that the knockdown of *CLEC3B*, another marker of the c3-ASC subpopulation, could significantly attenuate the fibrogenesis of ASCs from patients in vitro (Fig. 5). Altogether, we identified a potential cellular and molecular target for preventing or treating adipose tissue fibrosis in lymphoedema.

We found a striking depletion of the anti-inflammatory macrophages, i.e., the c6 *LYVE1*^+^ resident-like subpopulation, in the adipose tissues of lymphedema (Figs. 2c and 4b). It has been reported that *LYVE1*^+^ macrophages contributed to the homeostasis of the aorta through the control of collagen deposition by smooth muscle cells, thus preventing arterial stiffness^46^. In addition, our analysis revealed a perivascular ligand–receptor interaction module among ASCs, macrophages, and vascular endothelial cells in adipose tissues (Fig. 7), and found that ASCs were the predominant source of the macrophage colony-stimulating factor CSF1 (Supplementary Fig. S15a). These results reflect the close relationship between macrophages and ASCs in adipose tissues. The depletion of macrophages may contribute to the pathological changes in ASCs in lymphedema. Since the expression of *CSF1* in ASCs was even significantly higher in lymphedema than in healthy controls (Supplementary Fig. S15b), we reason that the mechanism underlying the depletion of macrophages, especially for the *LYVE1*^+^ macrophages, may not be due to pathological changes in ASCs. However, the precise mechanism remains to be explored. It has been reported that targeting immune cell subpopulations, such as CD4^+^ helper T cells^4^, was effective for alleviating lymphedema. We proposed that transplantation of *LYVE*^*+*^ anti-inflammatory macrophages could serve as a cellular therapy for cancer-associated lymphedema. Given the experimental difficulties of transplantation of *LYVE*^*+*^ macrophages, we tried to increase the ratio of M2/M1 macrophages (0.76 in CASE versus 2.03 in CTRL estimated by us) by inhibiting the pro-inflammatory M1 macrophages that highly expressed *TREM1*. We proved that the pharmacological blockage of Trem1 using mLR12 could significantly alleviate the lymphedema in a mouse tail model (Fig. 6). These results highlight the importance of anti-inflammatory therapies in lymphedema.

In summary, we provided a comprehensive analysis of the cellular heterogeneity, lineage-specific regulatory changes, and intercellular communication alterations of the SVF in adipose tissues from cancer-associated lymphedema at a single-cell resolution. Our study revealed lymphedema-associated cell subpopulations and a strong depletion of LYVE^+^ anti-inflammatory macrophage in lymphedema. We demonstrated that the knockdown of *CLEC3B* could significantly attenuate the fibrogenesis of ASCs from patients, and thus *CLEC3B* could serve as a potential target for alleviating adipose tissue fibrosis in lymphedema. In vivo experiments in mice suggested that pharmacological blockage of TREM1 using LR12 could serve as a promising medical therapy for treating lymphedema.

## Materials and methods

### Ethics approval

All human subject recruitments, tissue sampling procedures, and animal experiments have been approved by the ethics committee of Peking Union Medical College Hospital (JS-2528). Each subject provided written informed consent. All applicable institutional and/or national guidelines for the care and use of animals were followed.

### Adipose tissue specimen preparation and liposuction

Adipose tissue specimens were obtained from the affected thighs of five female patients with secondary lymphoedema (stage III) following surgical intervention for cervical cancer. As a control group, liposuction specimens from the thighs of four healthy female donors were collected during surgery for cosmetic purposes. The liposuction procedures used for both groups were the same and the surgeries of both groups were performed in the same division of plastic surgery. General anesthesia was done before liposuction. In total, 1000 ml tumescent solution containing 0.025% lidocaine and 1:1,000,000 epinephrine was injected into the thigh. Satisfactory infiltration of the tumescent solution was indicated by issue blanching and moderate tension. Liposuction was performed with 3-mm blunt-tip cannulas and 20 ml syringes in the deep fat layer. The syringes were stood up for 10 min to separate adipose from a tumescent fluid. Harvested adipose tissues were transferred into 50-ml sterile centrifuge tubes containing 20 ml Dulbecco’s Modified Eagle’s Medium (10569044, Gibco) cell culture medium, and transported on ice.

### SVF isolation and cell viability assay

Each fresh specimen was subjected to SVF isolation. Briefly, each specimen was washed several times with Hank’s balanced salt solution (14025126, Gibco). Subsequently, it was digested with 0.15% collagenase I (17100017, Gibco) supplied with 4% Penicillin-Streptomycin (15140122, Gibco) at 37 °C for 30 min. The pellet was resuspended in high-glucose Dulbecco’s Modified Eagle’s Medium (10569044, Gibco) with 10% fetal bovine serum (10099141, Gibco), filtered through a 100-μm strainer, and then centrifuged at 4 °C for 5 min. The obtained cell suspensions were resuspended in Hank’s balanced salt solution, and red blood cell lysis buffer was added at room temperature for 5 min to remove red blood cells. Then, it was centrifuged again, resuspended in Hank’s balanced salt solution with 0.04% bovine serum albumin (A1933-5G, Sigma), and filtered through a 40-μm strainer. Finally, the cells were centrifuged and resuspended in Dulbecco’s Phosphate Buffered Saline without Ca^2+^ and Mg^2+^ (14190144, Gibco). The obtained single-cell suspension was incubated with an equal volume of AOPI Staining Solution (Logos Biosystems). Cell concentration and viability were assayed using a LUNA-FL Fluorescence Cell Counter (Logos Biosystems). The cell viability of all samples subjected to scRNA-seq reached more than 90%.

### Animal model and treatment

To model lymphedema, we applied a widely used mouse tail model as previously described^4^. Briefly, the superficial and deep lymphatic channels of the tails of adult male (4–6-week-old, C57) mice were disrupted by a skin incision. Following the surgery, the mice were allowed to recover for 2 weeks and then randomized to scramble (*n* = 5) or treatment (*n* = 5) groups. To pharmacologically inhibit Trem1, a dodecapeptide, murine LR12 (LQEEDTGEYGCV; mLR12)^57^ was chemically synthesized (SBS Genetech, China) and COOH terminally amidated. The endotoxin was below 0.5 EU/mg. Starting from 2 weeks after the incision, mice were treated daily by intraperitoneal injection of mLR12 (5 mg/kg) for 6 weeks. As a control, mice were intraperitoneally injected daily with mLR12-scramble peptides. The diameter of the tail (1 cm distal to the incision) was measured every 3 days for 6 weeks. Tail tissue samples were collected 6 weeks after the surgery for subsequent assays.

### scRNA-seq

Freshly isolated SVF cells of each sample were subjected to scRNA-seq capture separately. Chromium Single-Cell 3' Reagent Kit v3 chemistry (10× Genomics, USA) was used for single-cell Gel Beads-in-Emulsion (GEM) generation, post-GEM-RT cleanup, barcoding, cDNA amplification, and cDNA library construction according to the manufacturer’s protocol. The NovaSeq 6000 system (Illumina, USA) was utilized to sequence the libraries.

### scRNA-seq data preprocessing

Sample demultiplexing, barcode processing, and unique molecular identifier (UMI) counting were performed using the official software Cell Ranger v3.0.2. Briefly, the pipeline “cellranger mkfastq” was used to demultiplex the raw base call files generated by the sequencers into reads in FASTQ format. Subsequently, a gene-barcode matrix for each library was generated using the pipeline “cellranger count” pipeline. Lastly, the pipeline “cellranger aggr” was used to concatenate the gene-cell UMI count matrices of all samples into one matrix. The concatenated gene-cell barcode matrix was imported into Seurat v3.1.0 for data preprocessing. Genes with counts in fewer than 3 cells were filtered out to exclude genes detected from random noise. Cell outliers (< first quartile − 1.5 × interquartile range or > third quartile + 1.5 × interquartile range) were filtered out according to expressed genes number, UMI count sum, and the proportion of mitochondrial genes to exclude poor-quality cells. We also filtered out doublets using the predictions by Scrublet^58^. We also excluded the cells enriched in hemoglobin gene expression. We normalized the sum of the UMI counts for each cell to 10,000 and then log-transformed the data. For each sample, the “FindVariableFeatures” function of Seurat was used to select 2000 features (genes). All the datasets were integrated via canonical correlation analysis implemented in Seurat to correct for potential batch effects. The mitochondrial gene proportion, S phase score, G2M phase score, and UMI count were regressed out with linear models to mitigate the effects of uninteresting sources of variation.

### Dimensional reduction and clustering of the scRNA-seq data

Linear dimensional reduction of the scRNA-seq data was performed through principal component analysis (PCA). A neighborhood graph of the cells was computed using the first 30 PCA components. Using UMAP^59^, the neighborhood graph was embedded in a two-dimensional space. The cells were clustered using Louvain clustering (resolution = 0.6) implemented in Seurat.

### Differential expression and functional enrichment analysis

The likelihood-ratio test (test.use: “bimod”) implemented in the function “FindMarkers” of Seurat was utilized to detect differentially expressed genes of the scRNA-seq data between two groups. The significance threshold was set to a log2-fold change > 0.25 and an adjusted *P* value < 0.05. The differential expression of candidate targets was confirmed by using a “pseudobulk” method^44^ (“edgeR-lrt”) implemented in the R package edgeR under default settings, which aggregates read counts across the single cells of the scRNA-seq data and accounts for variations among biological replicates. Functional enrichment analyses of a gene list were carried out using ClueGO^60^ with an adjusted *P* value < 0.05.

### Gene set enrichment analysis

Signal2Noise (the difference in means between CASE and CTRL scaled by the standard deviation) was applied to pre-rank all the expressed genes. The ranked gene list was then imported into the software GSEA (version: 4.0.1)^61^. The statistical significance cutoff was set to an FDR *q* value < 0.05. The precompiled REACTOME pathways in MSigDB (version: 7.0)^62^ were applied. We visualized the analysis results using the EnrichmentMap plugin of Cytoscape (version: 3.7.0).

### Differential proportion analysis

A permutation-based statistical test (differential proportion analysis) was performed as described previously^29^ to determine whether the change in relative proportions was expected by chance. The statistical significance threshold was set to a *P* value with Bonferroni correction < 0.05.

### Differential regulatory network analysis

Using the method implemented in bigScale2^43^, the single-cell transcriptomic data was used to build gene regulatory networks and comparative analysis was performed between the two groups. Briefly, the “compute.network” function (clustering = “direct”, quantile. p = 0.90) was used to infer gene regulatory networks separately for the CASE and CTRL. Genes encoding mitochondrial proteins or ribosomal proteins were excluded. Subsequently, using the function “homogenize.networks”, the number of edges was homogenized throughout the inferred networks. Lastly, using the function “compare.centrality”, we identified the changes in node centralities (the relative importance of genes in the network) in the CASE. Four measures of centrality were considered, including betweenness, degree, pagerank, and closeness.

### Subpopulation-specific regulon analysis

We performed regulon analysis using the R package SCENIC^34^ to identify the master regulators driving the cellular heterogeneity among subpopulations. Briefly, coexpression modules that included a set of genes coexpressed with regulators were identified. Only the modules with significant motif enrichment of the regulators (referred to as regulons) were retained. Each cell was scored for the activity of each regulon. According to the average regulon activity scores of cells in the subpopulation, subpopulation-specific regulons were identified.

### Cell–cell communication analysis

CellPhoneDB 2.0^51^, which allows for statistically inferring lineage-specific interactions, was applied to analyze cell–cell communication according to the single-cell transcriptomic dataset. Briefly, ligand–receptor interactions were predicted according to the expression of a receptor by one lineage and a ligand by another. We only considered the ligands and receptors expressed in greater than 10% of the cells in any given cell type. To generate a null distribution for each ligand–receptor pair in each pairwise comparison between lineages, we randomly permuted the labels of all cells 1000 times and calculated the means of the average ligand-receptor expression in the interacting cell types. A *P* value for the likelihood of lineage specificity was ultimately obtained for each ligand–receptor pair.

### Knockdown of *CLEC3B* expression in ASCs

ASCs were cultured in complete medium, i.e., Dulbecco’s modified Eagle’s medium (11885084, Gibco) supplemented with 10% fetal bovine serum (10099141, Gibco) and 1% Penicillin-Streptomycin (15140122, Gibco), at 37 °C with 5% CO_2_. Before the transfection, the cells were trypsinized and counted. Then, the cells were diluted in an antibiotic-free culture medium, plated into 6-well plates, and incubated at 37 °C with 5% CO_2_ overnight. ON-TARGETplus Human CLEC3B siRNA Smartpool (L-019749-00-0005, Horizon) was synthesized to knockdown the expression of *CLEC3B*. ON-TARGETplus non-targeting pool was used as a negative control (Scramble siRNA, D-001810-10-05, Horizon). The ON-TARGETplus siRNA technology reduces off-targets through strand modifications and seed-region analysis on siRNA design. Cell transfection was carried out using the DharmaFECT 4 siRNA Transfection Reagent (T-2004-02, Dharmacon) according to the manufacturer’s instructions. The cells were plated in an antibiotic-free culture medium with 25 nM siRNA concentrations at 37 °C with 5% CO_2_. The culture medium was changed into an antibiotic-containing medium 24 h after transfection. The cells were collected at 48 h for mRNA analysis and 96 h for protein analysis.

### Bulk RNA-seq and data analysis

After the incubation for 48 h, the cells were harvested for total RNA extraction using TRIzol reagent (15596018, Invitrogen). A NEBNext Ultra RNA Library Prep Kit for Illumina (E7530L, NEB) was used for bulk RNA-seq library construction. The libraries were subjected to sequencing using the Illumina X Ten system. Following quality control with fastp^63^, the sequencing reads were subjected to transcript abundance quantification with kallisto^64^. Differentially expressed genes between groups were identified using sleuth^65^. The statistical significance cutoff was set to a *q* value of 0.05.

### RNA extraction and gene expression assay with qPCR

The total RNA from the cells or the mouse tail tissues was extracted with TRIzol reagent (15596018, Invitrogen). Single-stranded DNA was synthesized using PrimeScript Reverse Transcriptase (2680A, TaKaRa). qPCR was performed using the PowerUp^TM^ SYBR^TM^ Green Master Mix (A25742, Applied Biosystems). *GAPDH* was used as a reference gene. The relative levels of mRNAs were calculated by the 2^–ΔΔCt^ method. All primers used are listed below: *CLEC3B*, 5′-GCCTCTTCTCCCTCCTGAC-3′ (forward) and 5′-ATCTTTGTGTTCACAACATCTTTCT-3′ (reverse); *PRG4*, 5′-AGTTTCATCTCAAGAGCTTTCCTGT-3′ (forward) and 5′-GGTGATGTGGGATTATGCACTTCTG-3′ (reverse); *FN1*, 5′-GAGAATAAGCTGTACCATCGCAA-3′ (forward) and 5′-CGACCACATAGGAAGTCCCAG-3′ (reverse); *CCN2*, 5′-CAGCATGGACGTTCGTCTG-3′ (forward) and 5′-AACCACGGTTTGGTCCTTGG-3′ (reverse); *COL1A1*, 5′-GAGGGCCAAGACGAAGACATC-3′ (forward) and 5′-CAGATCACGTCATCGCACAAC-3′ (reverse); *GAPDH*, 5′-CTATAAATTGAGCCCGCAGCC-3′ (forward) and 5′-GCCCAATACGACCAAATCCGT-3′ (reverse); *Cd68*, 5′-TGTCTGATCTTGCTAGGACCG-3′ (forward) and 5′-GAGAGTAACGGCCTTTTTGTGA-3′ (reverse); *Tnf*, 5′-TAGCCCACGTCGTAGCAAAC-3′ (forward) and 5′-GCAGCCTTGTCCCTTGAAGA-3′ (reverse); *Il1b*, 5′-TGCCACCTTTTGACAGTGATGA-3′ (forward) and 5′-GCTCTTGTTGATGTGCTGCTG-3′ (reverse); *Gapdh*, 5′-TCGTCCCGTAGACAAAATGG-3′ (forward) and 5′-TTGAGGTCAATGAGGGGGTC-3′ (reverse).

### Immunoblot assay

Cultured ASCs were lysed on ice in NP-40 Lysis Buffer (P0013F, Beyotime) containing protease and phosphatase inhibitor. Lysate protein concentrations were determined with Micro BCA Protein Assay Kit (23235, Thermo Scientific). In total, 200–400 μg of protein per sample (*n* = 3) was used for western blotting analysis. Proteins were separated by 4%–12% Bis-Tris Bolt gels (NW04120BOX, Invitrogen) and transferred to pure nitrocellulose blotting membranes (66485, Pall Life Sciences). Membranes were blocked with 5% nonfat milk (P0216, Beyotime) or 3% bovine serum albumin (ST023, Beyotime) in Tris-Buffered Saline with Tween-20 (ST671, Beyotime) at room temperature for 1 h. Target proteins were incubated overnight at 4 °C with the following primary antibodies: CLEC3B (ab51883, Abcam), Type I Collagen (ab138492, Abcam), SMAD-2/3 (3102S, Cell Signaling Technology), and phosphorylated SMAD-2/3 (8828S, Cell Signaling Technology). HRP-conjugated corresponding secondary antibodies (ZB-2301/2305, ZSGB-BIO) were used at room temperature for 1 h, followed by chemiluminescent detection (P0018AM, Beyotime). Equal loading controls were indicated with GAPDH (2118L, Cell Signaling Technology). Densitometry analysis was performed by quantifying the intensity of the bands using the ImageJ software.

### Paraffin section preparation

Adipose tissues from patients or mouse tail tissues were isolated and fixed in 4% paraformaldehyde (DF0135, LEAGENE). The mouse tail tissues were decalcified in 10% neutral buffered EDTA (DD0002, LEAGENE) at 37 °C for 2 weeks. The adipose tissues and mouse tail tissues were dehydrated by ASP300S (Leica) and then were paraffin-embedded. The wax blocks were cut into 5-μm sections with RM2235 (Leica) for the subsequent staining. For all the staining experiments of mouse tail tissues, cross-sections of the mouse tails at almost the same level (located 1 cm distal to the surgical site) were used for both groups. The staining results were visualized on the Pannoramic SCAN (3DHISTECH) or the Vectra Polaris pathology imaging system (PerkinElmer).

### H&E staining

H&E staining was conducted using routine protocols on the Varistain Gemini automatic stainer (Thermo Fisher Scientific). Subcutaneous tissue thickness analysis was performed to measure the distance from the basal layer of the epidermis to the deep fascia. A total of three representative regions per section were analyzed using the ImageJ software.

### Sirius red staining

The sections were incubated with hematoxylin solution for 5 min, 0.1% Sirius red solution for 1 h, dehydrated in absolute alcohol, cleared in xylene, and mounted with neutral balsam. Collagen and non-collagen components were stained in red and yellow, respectively.

### Immunofluorescent staining of tissue sections and cultured cells

Tissue sections were deparaffinized, and then the antigen was repaired with EDTA solution (pH = 9.0) or sodium citrate (pH = 6.0) under high pressure for 2 min. Subsequently, the sections were blocked with 10% goat serum at room temperature for 1 h, followed by primary antibodies at 4 °C overnight. Sections were washed three times with Phosphate Buffered Saline, and then stained with Alexa Fluor 488 goat anti-rabbit (A-11008, Invitrogen) and Alexa Fluor 594 goat anti-mouse (ab150116, Abcam) secondary antibodies at room temperature for 40 min. The sections were sealed with 4′,6-diamidino-2-phenylindole (DAPI) mounting solution (ZLI-9557, ZSGB-BIO). The primary antibodies used for immunofluorescent staining included: PRG4 (ab28484, Abcam), CHI3L1 (ab77528, Abcam), CTHRC1 (ab85739, Abcam), CXCL14 (ab46010, Abcam), PDGFRα (MAB322, R&D), Cd11b (ab8878, Abcam), and Trem1 (bs-4886R, Bioss).

Cultured cells were fixed in 4% paraformaldehyde (DF0135, LEAGENE) for 10 min at room temperature, and permeabilized with 0.2% Triton X-100 (A110694-0100, Diamond) in Phosphate Buffered Saline for 20 min. Thereafter, cells were blocked with 3% bovine serum albumin (A1933-5G, Sigma) in Phosphate Buffered Saline for 1 h, followed by primary antibody Collagen-1 (ab138492, Abcam) at 4 °C overnight. Cells were then stained with Alexa Fluor 488 goat anti-rabbit (A-11008, Invitrogen) secondary antibody at room temperature for 1 h. DAPI (4083S, Cell Signaling Technology) was used for nuclear counterstaining. The cells were imaged using the Axio Vert.A1 Microscope (ZEISS). Mean fluorescence intensity was measured using the ImageJ software.

### Flow cytometry

SVF was resuspended in cell staining buffer (420201, BioLegend), and then incubated with the following cellular surface antibodies at room temperature in the dark for 30 min: PDGFRα (CD140a)-Phycoerythrin (323506, BioLegend) and CD55-Allophycocyanin (311312, BioLegend). The cells were washed twice with cell staining buffer to remove unbound antibodies. Flow cytometry data were acquired on the Accuri C6 flow cytometer (BD Biosciences) and analyzed using the FlowJo software (BD Biosciences).

## Supplementary information

Supplmentary figures S1–S15

Supplementary Table S1

Supplementary Table S2

Supplementary Table S3

Supplementary Table S4

Supplementary Table S5

Supplementary Table S6

Supplementary Table S7

Supplementary Table S8

Supplementary Table S9

Supplementary Table S10

Supplementary Table S11

## Data Availability

Datasets related to this article can be found at https://ngdc.cncb.ac.cn/gsa-human/browse/HRA000901, hosted at Genome Sequence Archive.
