# Supplementary material for: Single-cell RNA sequencing of subcutaneous adipose tissues identifies therapeutic targets for cancer-associated lymphedema
Source: Cell Discov. 2022 Jun 21;8:58. doi: 10.1038/s41421-022-00402-5 (PMC9209506; doi:10.1038/s41421-022-00402-5)
Supplement: Supplementary file 1 — Supplmentary figures S1–S15 [file 41421_2022_402_MOESM1_ESM.pdf]

Supplementary information

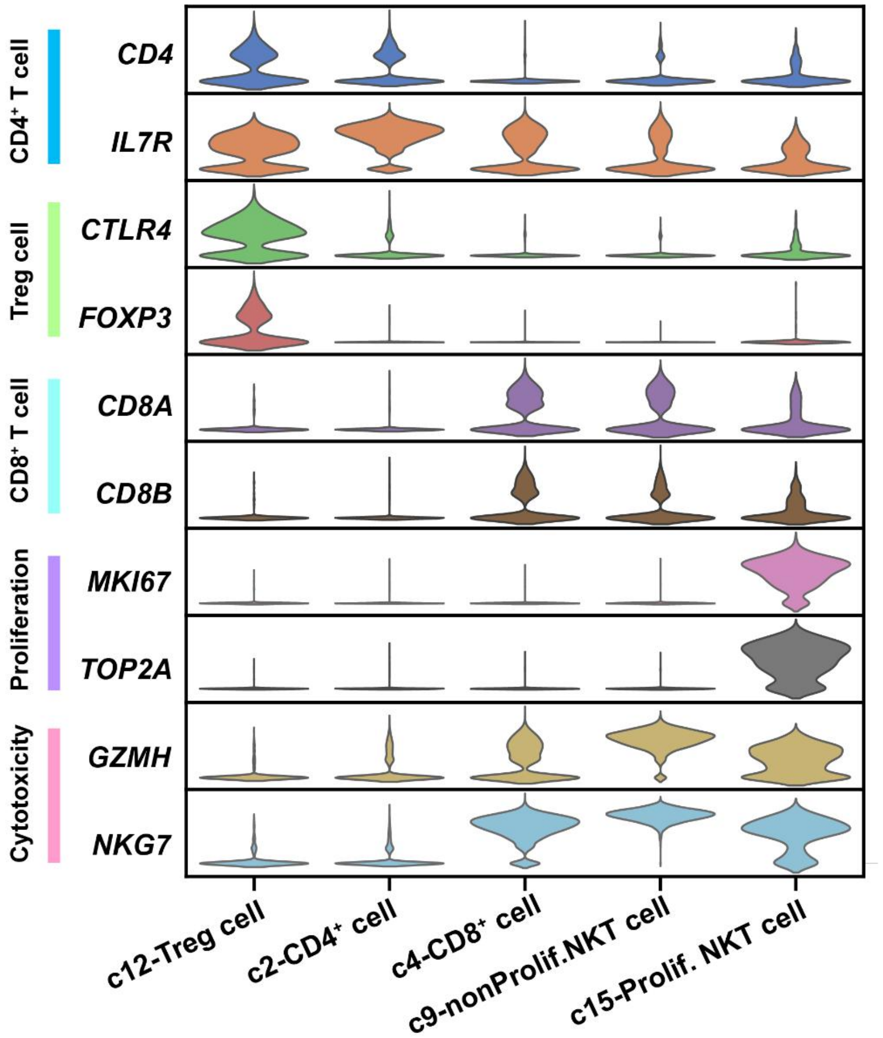

Fig. S1 Expression of markers for CD4$^+$ T cells, Treg cells, CD8$^+$ T cells, proliferation, and cytotoxicity in the clusters of T cells and NKT cells.

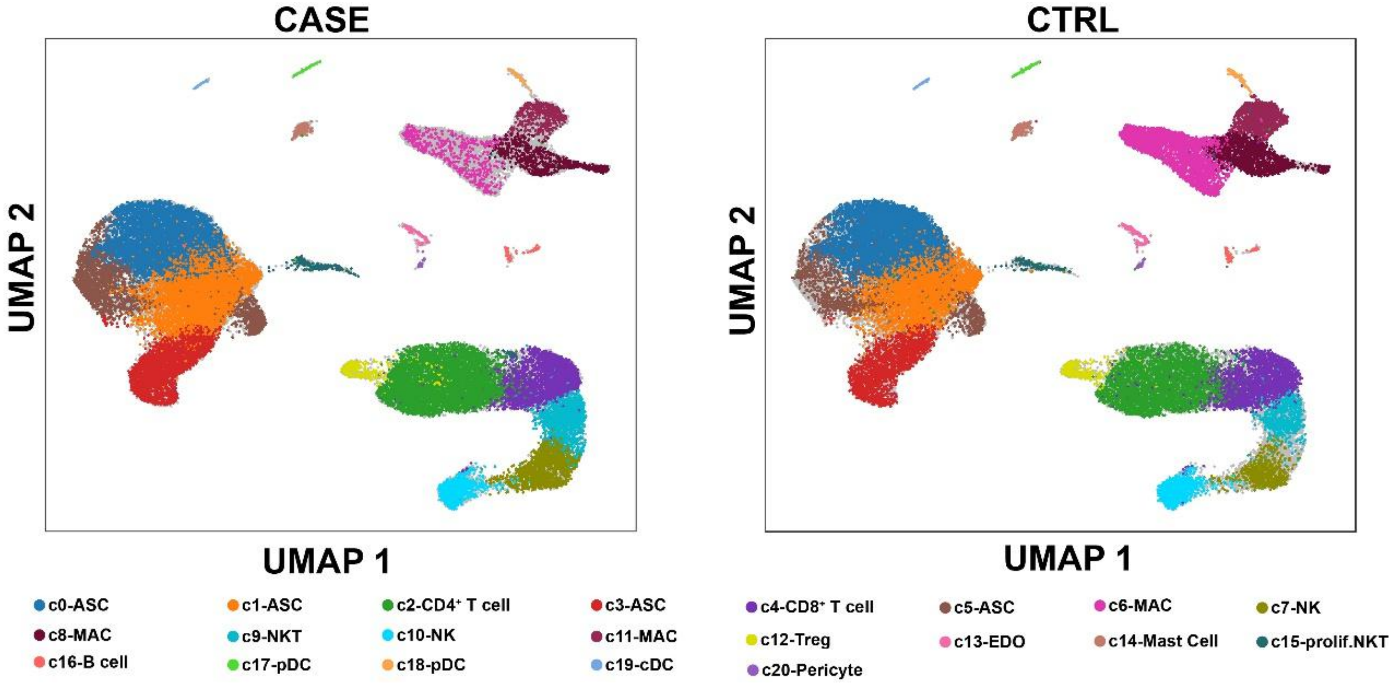

Fig. S2 UMAP plots showing the distribution of cells from each group in each cluster. The CASE group was randomly downsampled to make the two groups have an equal number of cells.

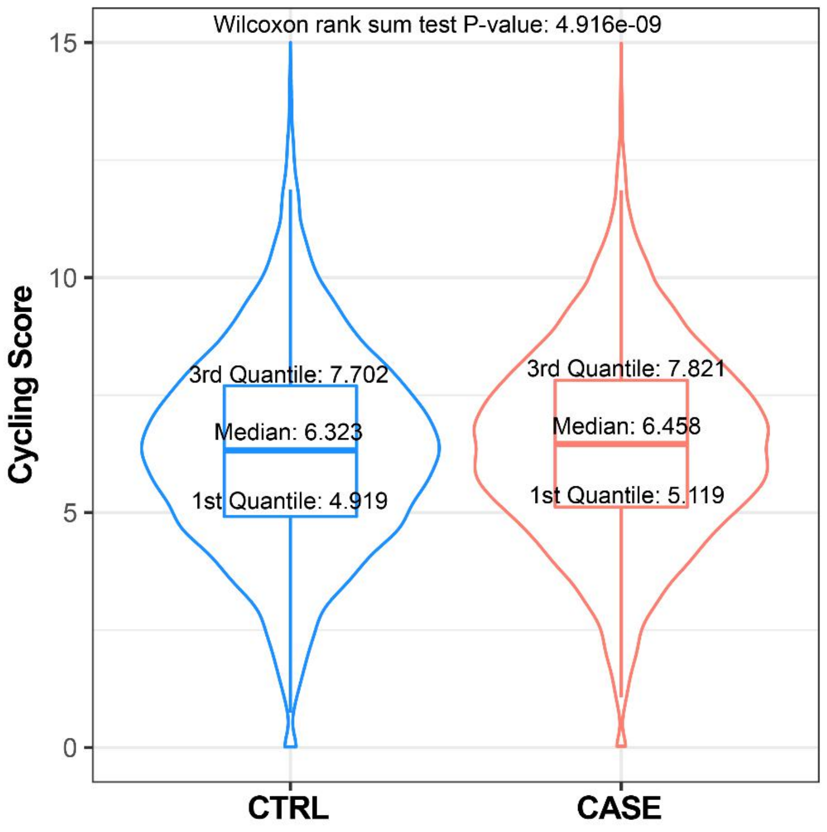

Fig. S3 The distribution of cycling scores of ASCs in CASE and CTRL. The cycling score is defined as the sum of the expression of a group of cycling genes.

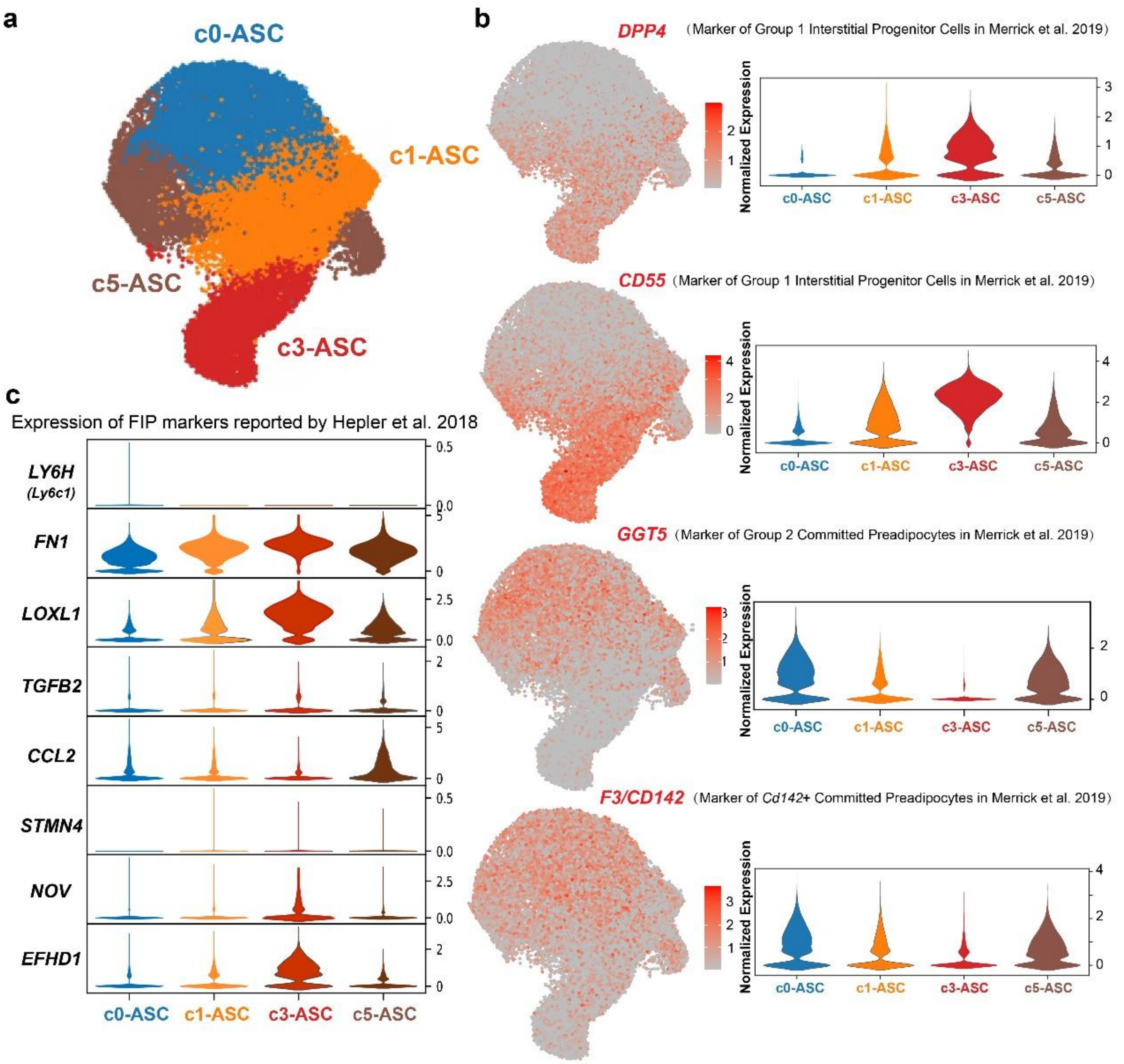

Fig. S4 Expression of the markers of ASC subpopulations reported in previous representative studies. **a** UMAP plot showing the distribution of the four ASC subpopulations identified in the present study. **b** Expression of the markers of human ASC subgroups reported by Merrick et al. (2019, Science) across the four ASC subpopulations identified in the present study. SVF from the abdominal subcutaneous adipose tissue of one female was analyzed in Merrick et al.'s study and two subgroups were reported: Group 1 and Group 2. *DPP4* and *CD55* are the markers of multipotent interstitial progenitor cells (Group 1). *GGT5* is the marker of committed preadipocytes (Group 2, restricted to the adipocyte lineage). *F3/CD142* is the marker of a subcluster of committed preadipocytes, which was identified in mouse adipose tissue. But no significant difference in *CD142* expression was found between Group 1 and Group 2 ASCs from human adipose tissues. However, in our dataset, the expression levels of CD142 exhibited a significant difference between committed preadipocytes (e.g., c0-ASC) and interstitial progenitor cells (e.g., c3-ASC). **c** Expression of representative markers of fibro-inflammatory progenitors (FIPs) reported by Hepler et al. (2018, Elife) across the four ASC subpopulations identified in the present study. FIPs were identified in visceral white adipose tissue of adult mice by Hepler et al.. The subpopulation c3-ASC expressed high levels of some FIP markers (for example, fibrosis-associated marker, *FN1,* and *LOXL1*) but exhibited very low expression for other markers (for example, inflammation-associated marker *CCL2*). Thus, c3-ACSs do not correspond to

FIPs.

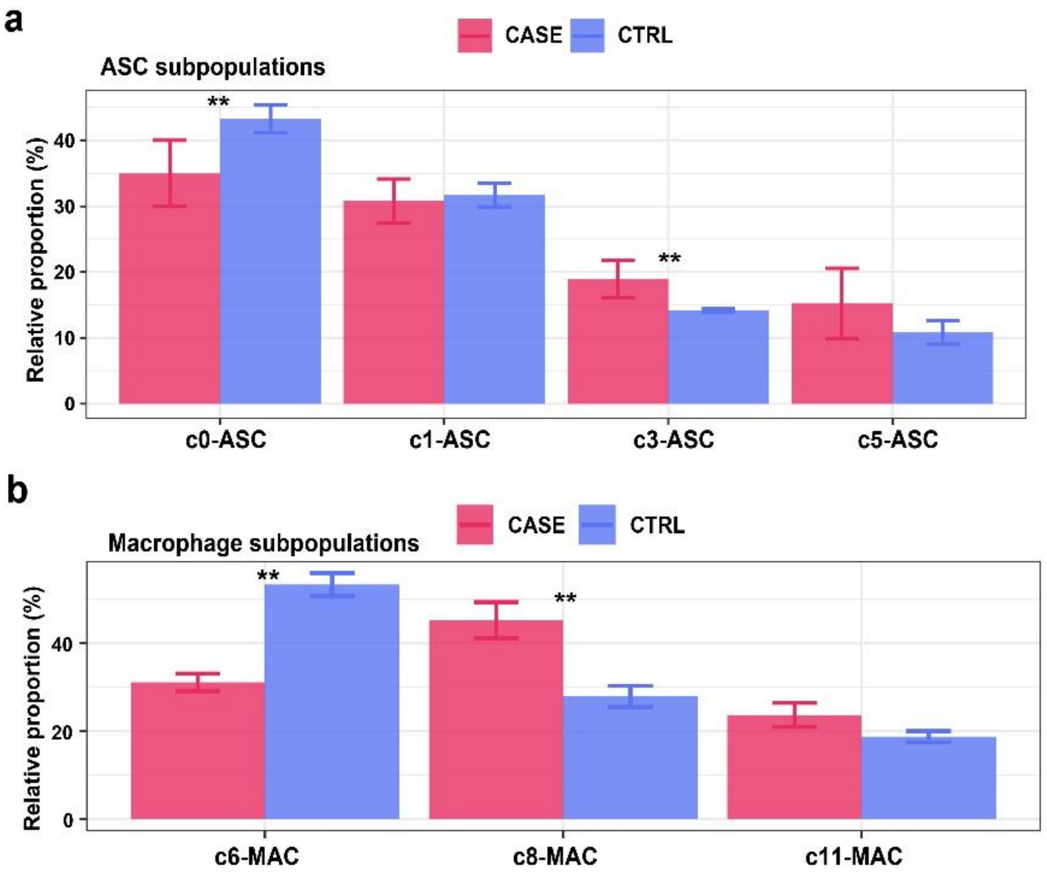

Fig. S5 Relative proportions of each subpopulation in the ASC or macrophage lineages under both conditions. a Relative proportions of each ASC subpopulation in ASCs. **b** Relative proportions of each macrophage subpopulation in macrophages. In a and b, the sum of relative proportions of all subpopulations in each sample equals 100%. A permutation-based statistical test (differential proportion analysis) was performed. The error bar represents mean ± SE. **: Bonferroni-corrected P value < 0.01. CASE: cancer-associated lymphedema; CTRL: healthy control.

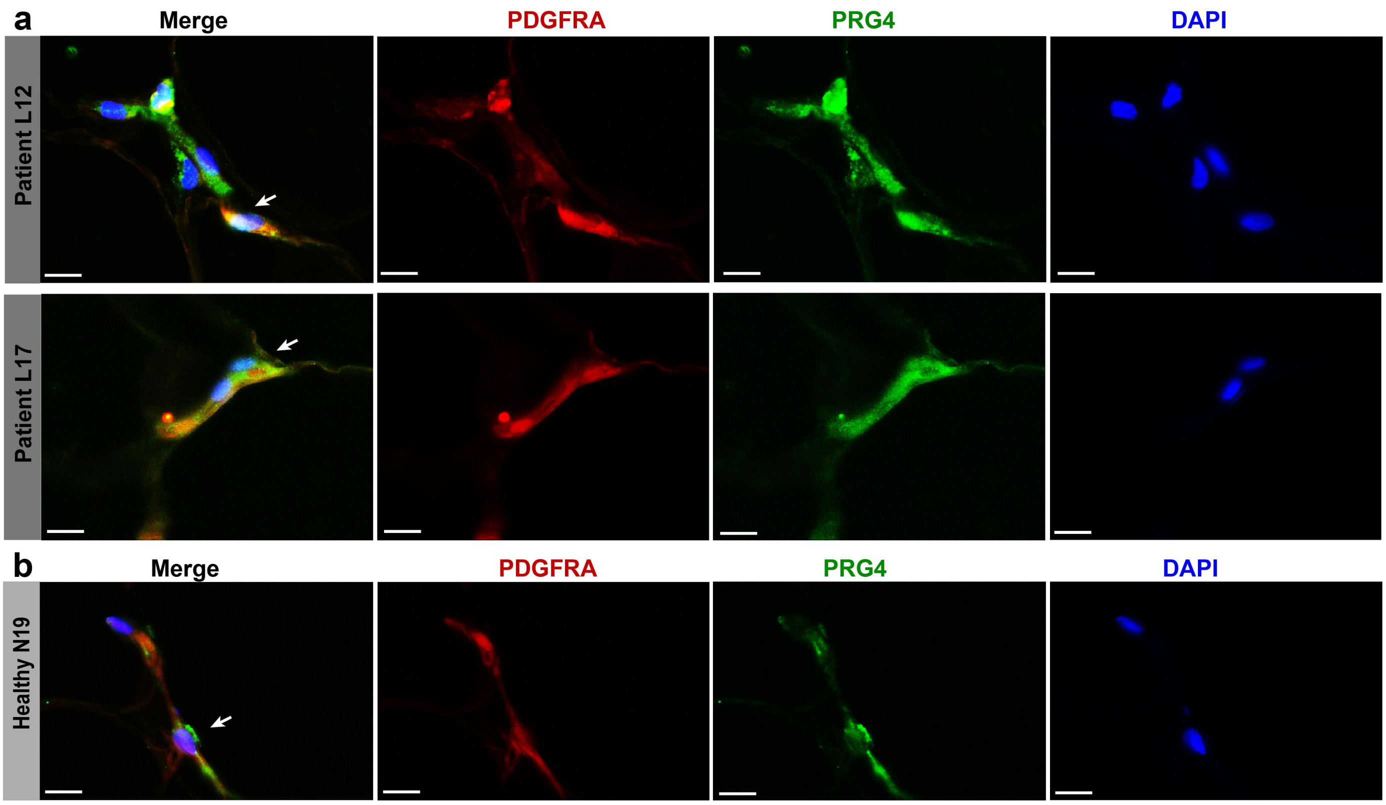

Fig. S6 Immunofluorescence staining showing the presence of the c3-ASC subpopulation in the subcutaneous adipose tissues of lymphedema patients and healthy donors. a Staining results in adipose tissue sections of two patients. **b** Staining results in adipose tissue sections of one healthy donor. The staining results of other subjects are shown in Fig. 3e-3f. Arrows indicate the $PDGFRA^{+}$ $PRG4^{+}$ ASCs cells. Scale bar: 10 μm.

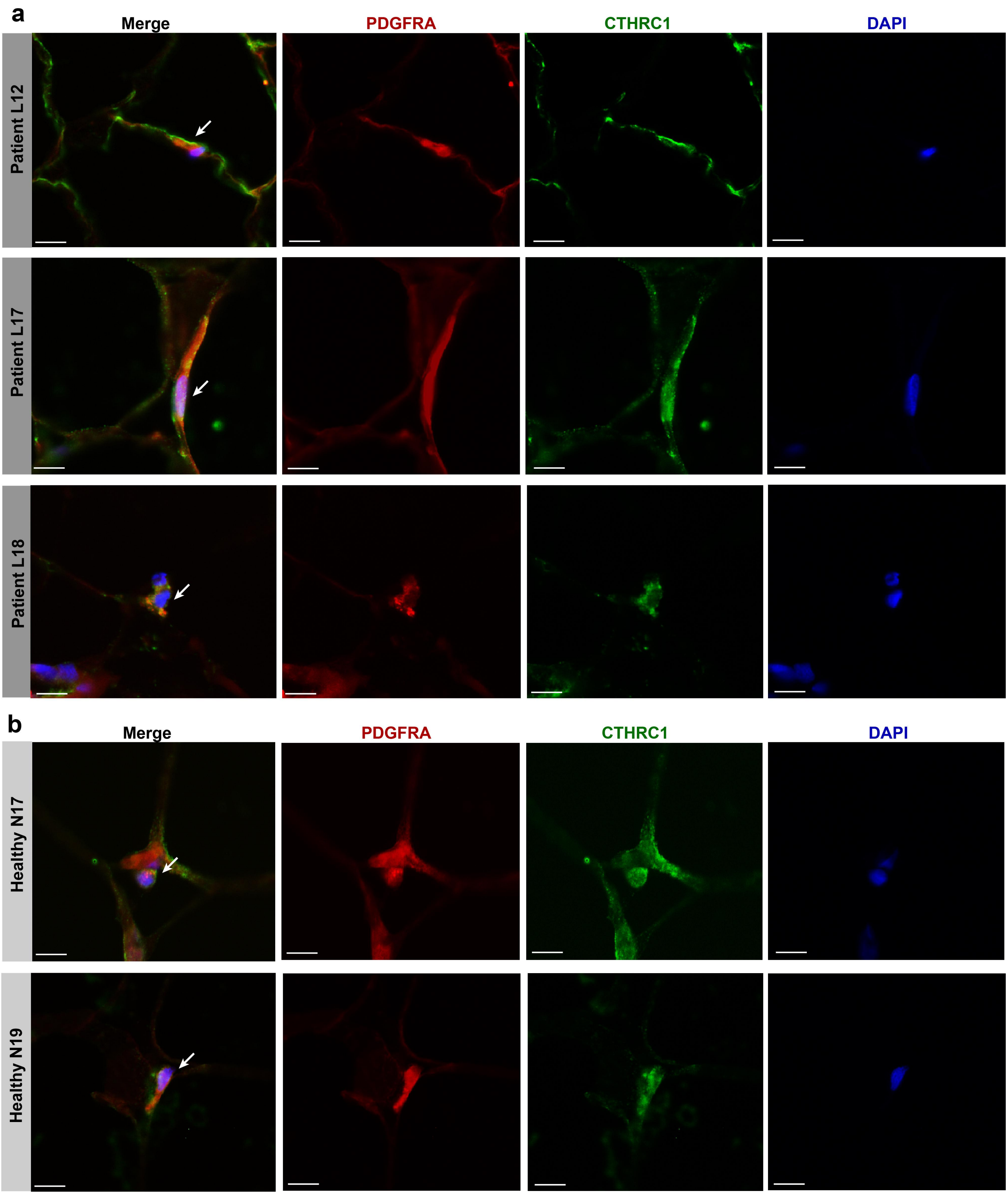

Fig. S7 Immunofluorescence staining showing the presence of the c1-ASC subpopulation in the subcutaneous adipose tissues of lymphedema patients and healthy donors. a Staining results in adipose tissue sections of three lymphedema patients. **b** Staining results in adipose tissue sections of two healthy donors. Arrows indicate the PDGFRA^{+} CTHRC1 ^{high} ASCs cells. Scale bar: 10 µm.

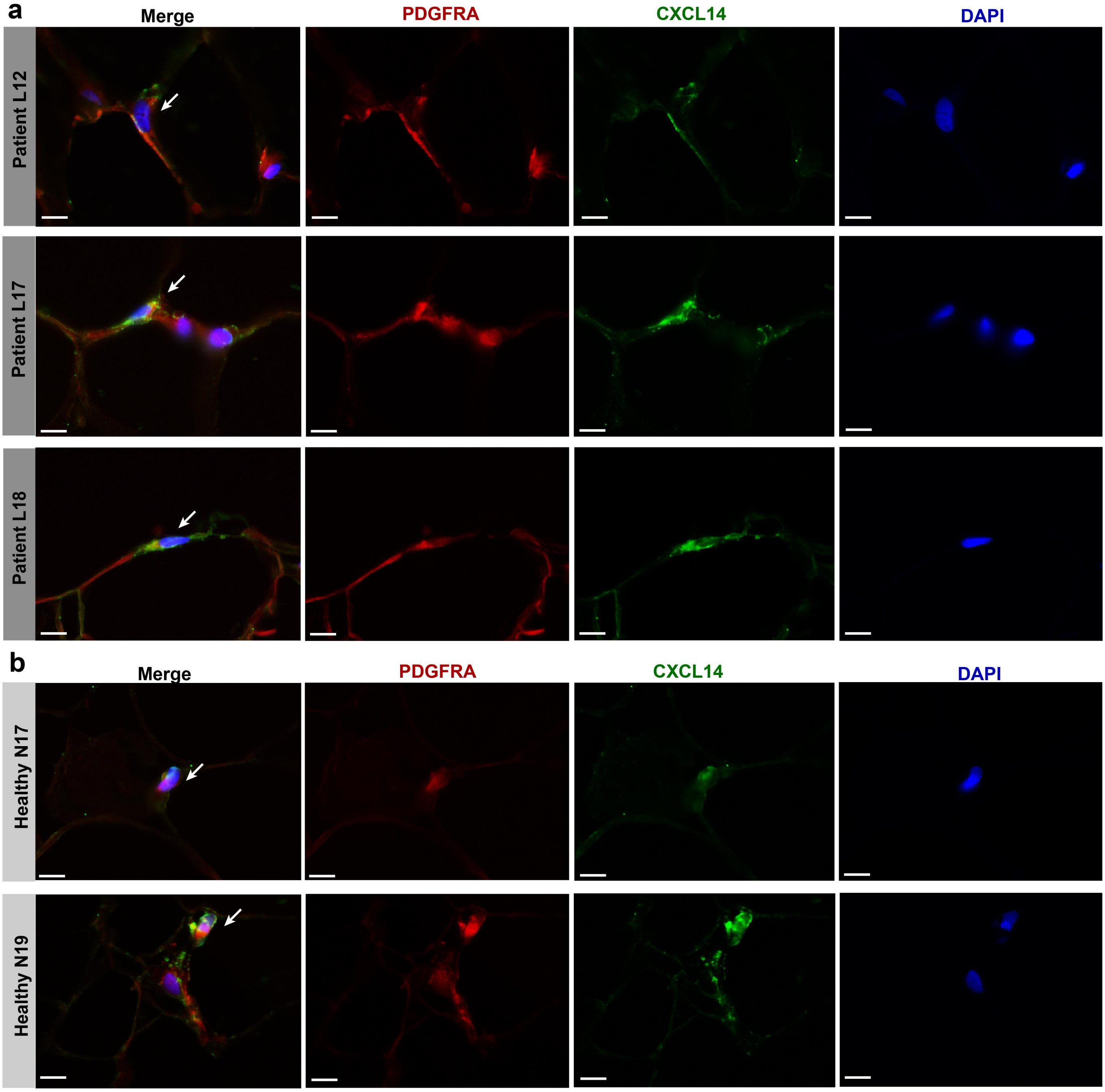

Fig. S8 Immunofluorescence staining showing the presence of the c0-ASC subpopulation in the subcutaneous adipose tissues of lymphedema patients and healthy donors. a Staining results in adipose tissue sections of three lymphedema patients. **b** Staining results in adipose tissue sections of two healthy donors. Arrows indicate the PDGFRA^{+} CXCL14 ^{high} ASCs cells. Scale bar: 10 μm.

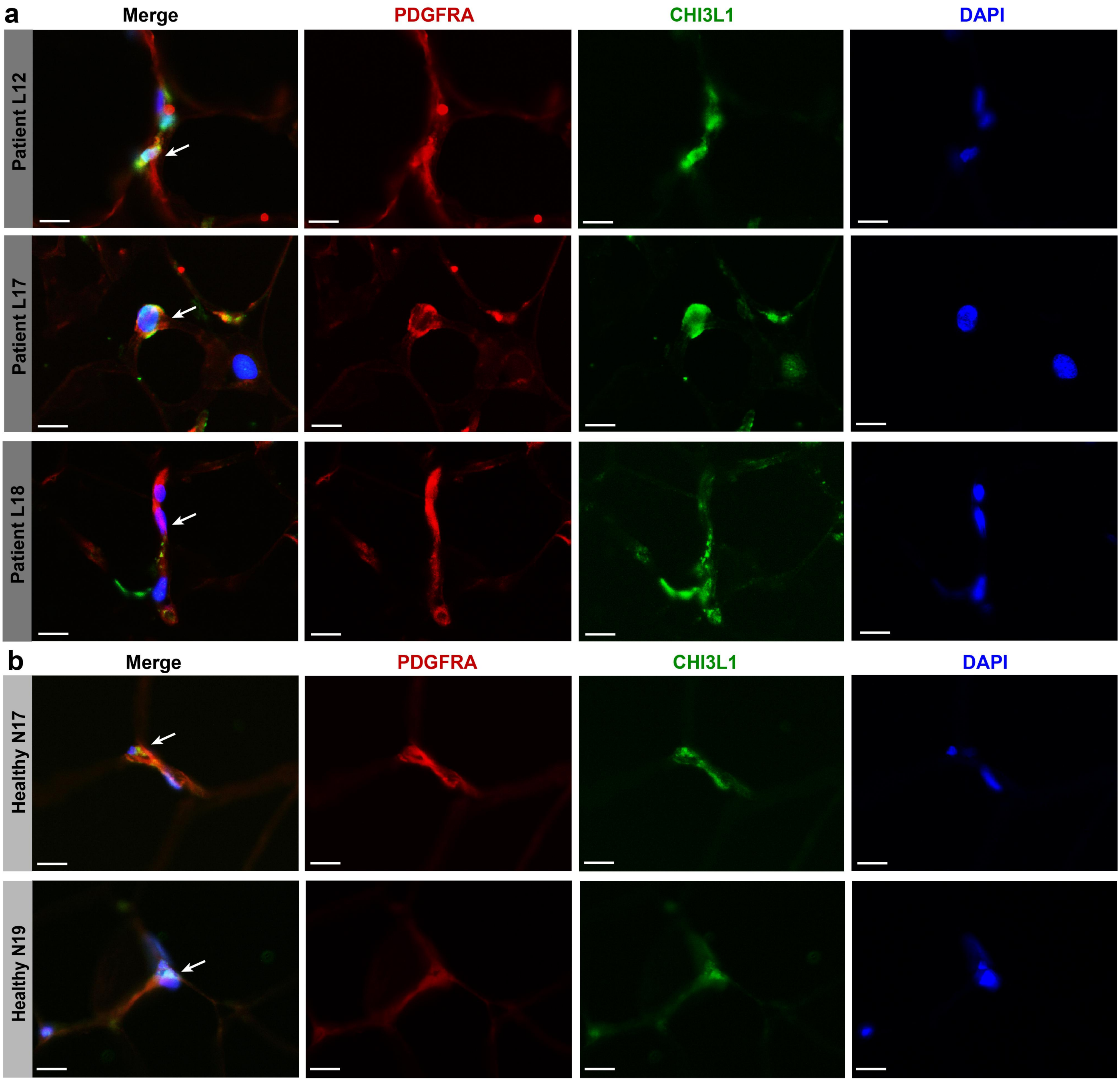

Fig. S9 Immunofluorescence staining showing the presence of the c5-ASC subpopulation in the subcutaneous adipose tissues of lymphedema patients and healthy donors. a Staining results in adipose tissue sections of three lymphedema patients. **b** Staining results in adipose tissue sections of two healthy donors. Arrows indicate the PDGFRA^{+} CHI3L1 ^{high} ASCs cells. Scale bar: 10 μm.

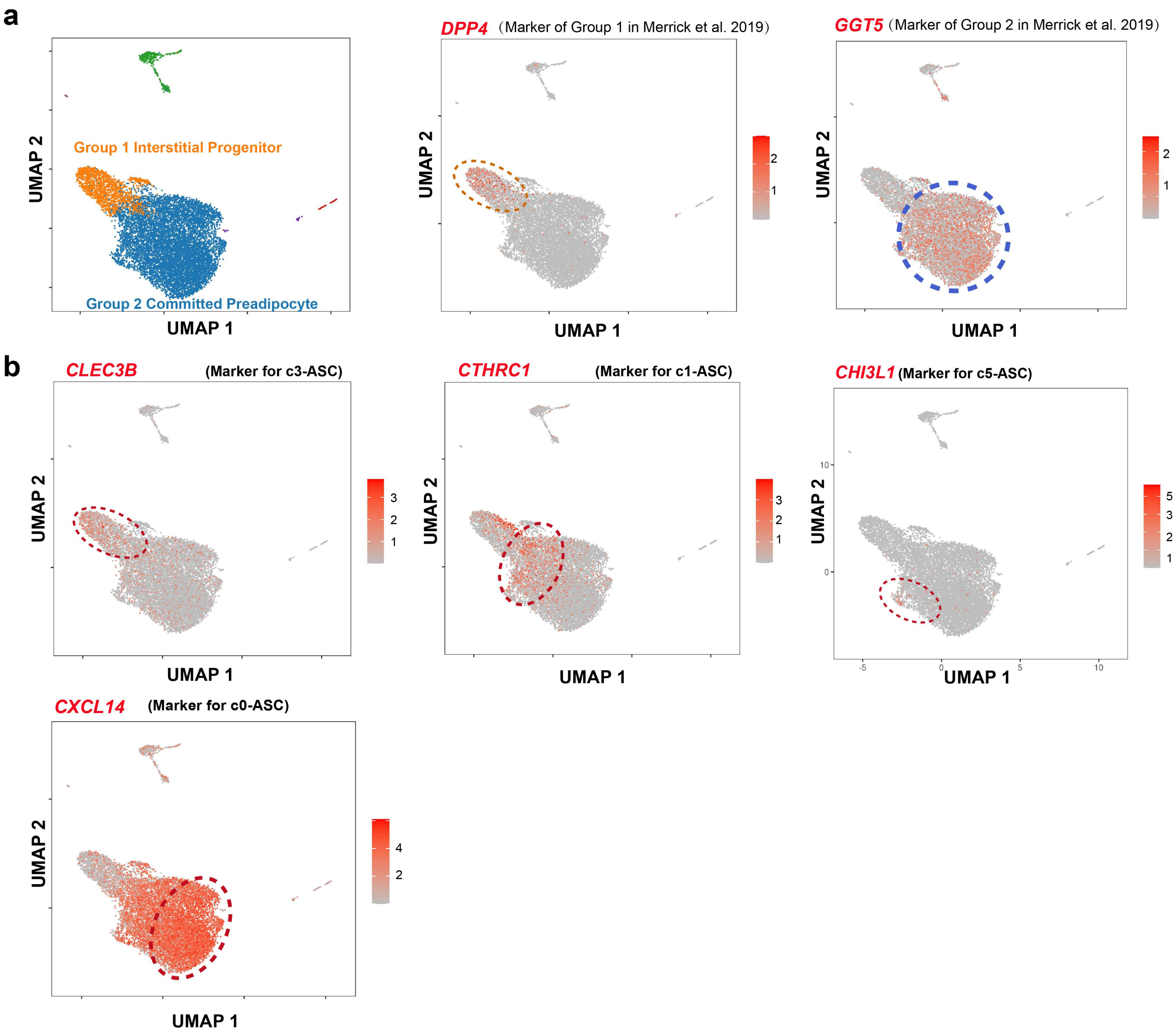

Fig. S10 The expression of the markers for the four ASC subpopulations we identified in the data of Merrick et al.'s study (2019, Science). a UMAP plot showing the two ASC groups reported in the original publication. The dataset (GEO accession number: GSM3717979) was reanalyzed (resolution = 0.1). **b** UMAP plot showing the presence of the four ASC subpopulations in Merrick et al.'s data. *CLEC3B* ^{high} cells (c3-ASC markers) are mainly distributed in Group 1. Concordant with the intermediate states of c1-ASCs, *CTHRC1* ^{high} cells (c1-ASC marker) are mainly distributed in the border region between Group 1 and Group 2. *CHI3L1* ^{high} cells (c5-ASC marker) formed a distinct cluster that belong to a subset of Group 2 cells. *CXCL14* ^{high} cells (c0-ASC marker) represent a more committed state of Group 2 preadipocytes.

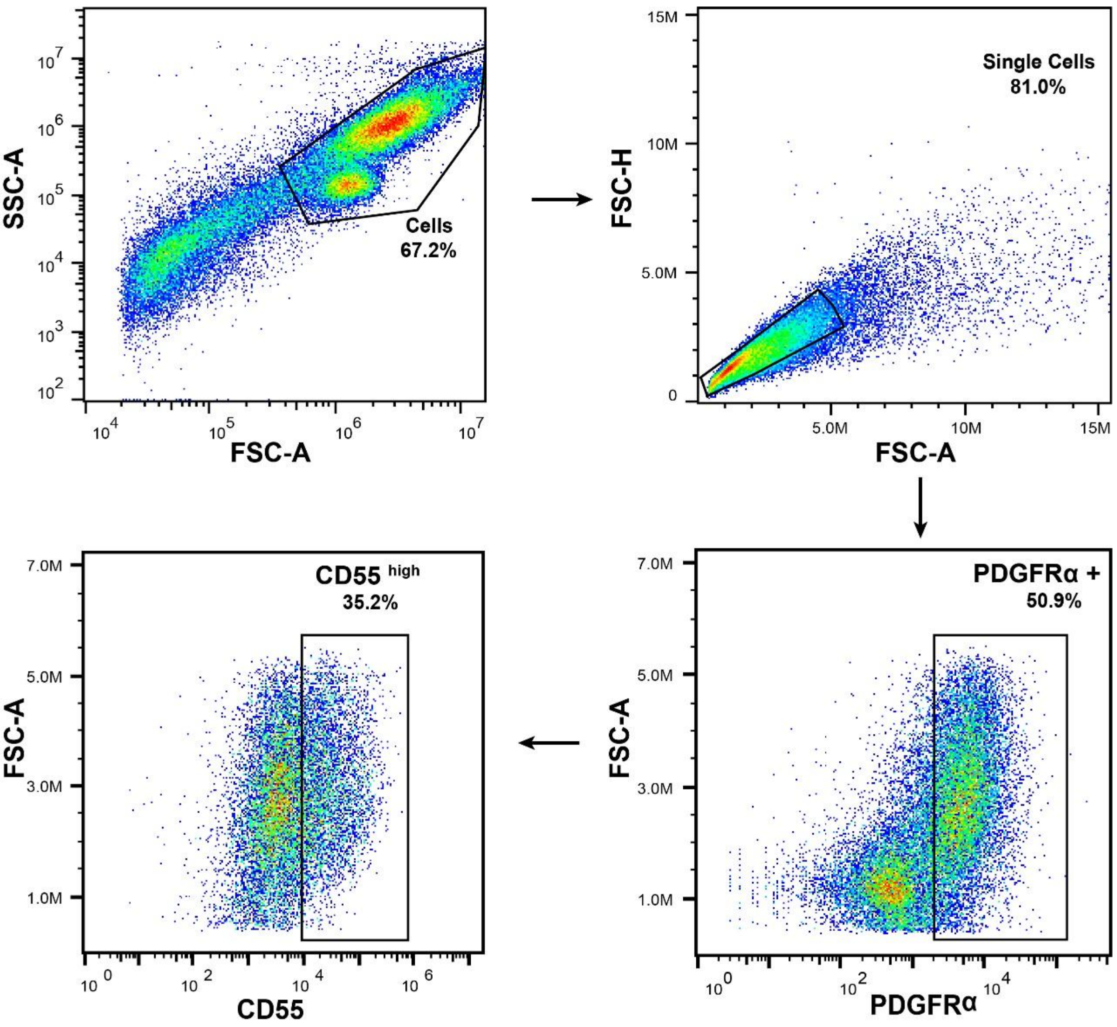

Fig. S11 Flow cytometry gating strategy for analyzing the proportion of CD55 ^{high} cells in ASCs (PDGFRα^{+}) cells. Debris and doublets were excluded based on forward and side scatter data. Unstained cells were used to control the gating strategy. FSC-A: forward scatter-area; FSC-H: forward scatter-height; SSC-A: side scatter-area.

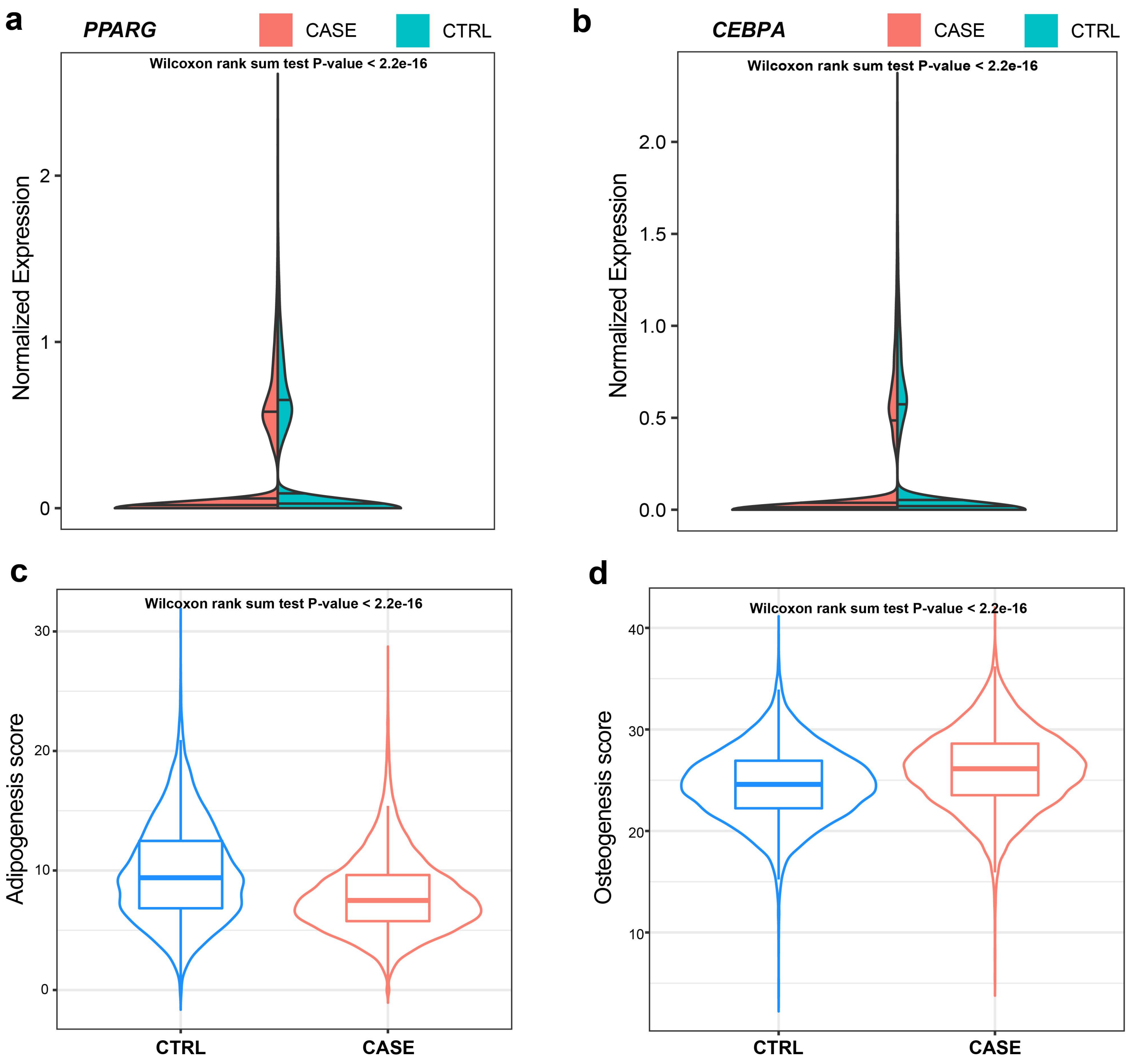

Fig. S12 Decreased adipogenesis and increased osteogenesis of ASCs in lymphedema. a The expression of *PPARG*, the master regulator of adipogenesis, was significantly decreased in CASE compared to CTRL. **b** The expression of *CEBPA*, another key regulator of adipogenesis, was significantly decreased in CASE compared to CTRL. **c** Decreased adipogenesis in CASE compared to CTRL. The adipogenesis score is defined as the sum of the expression of a curated list of genes involved in adipogenesis, including *ACACA, ANGPTL4, APOE, CD36, CEBPA, CEBPB, CEBPD, FASN, INSR, PPARG, SREBF1, IGF1, PLIN2, ADIPOQ, AOC3, AQP7, CITED1, FABP4, LEP, LPL, PCK1, SCD, SLC27A1, SLC2A4, SLCO2A1,* and *UCP1*. **d** Increased osteogenesis in CASE compared to CTRL. The osteogenesis score is defined as the sum of the expression of a curated list of genes involved in osteogenesis, including *BMP2, COL11A1, COL9A2, COMP, FGFR3, HAPLN1, IHH, PTCH1, SOX5, SOX6, SOX9, TNFSF11, WNT11, WNT4, ACAN, BMP7, CD151, COL10A1, COL2A1, COL4A1, COL9A3, DMP1, EPYC, IBSP, MEF2C, MMP3, PAPLN, PRG4, RUNX3,* and *MIA*.

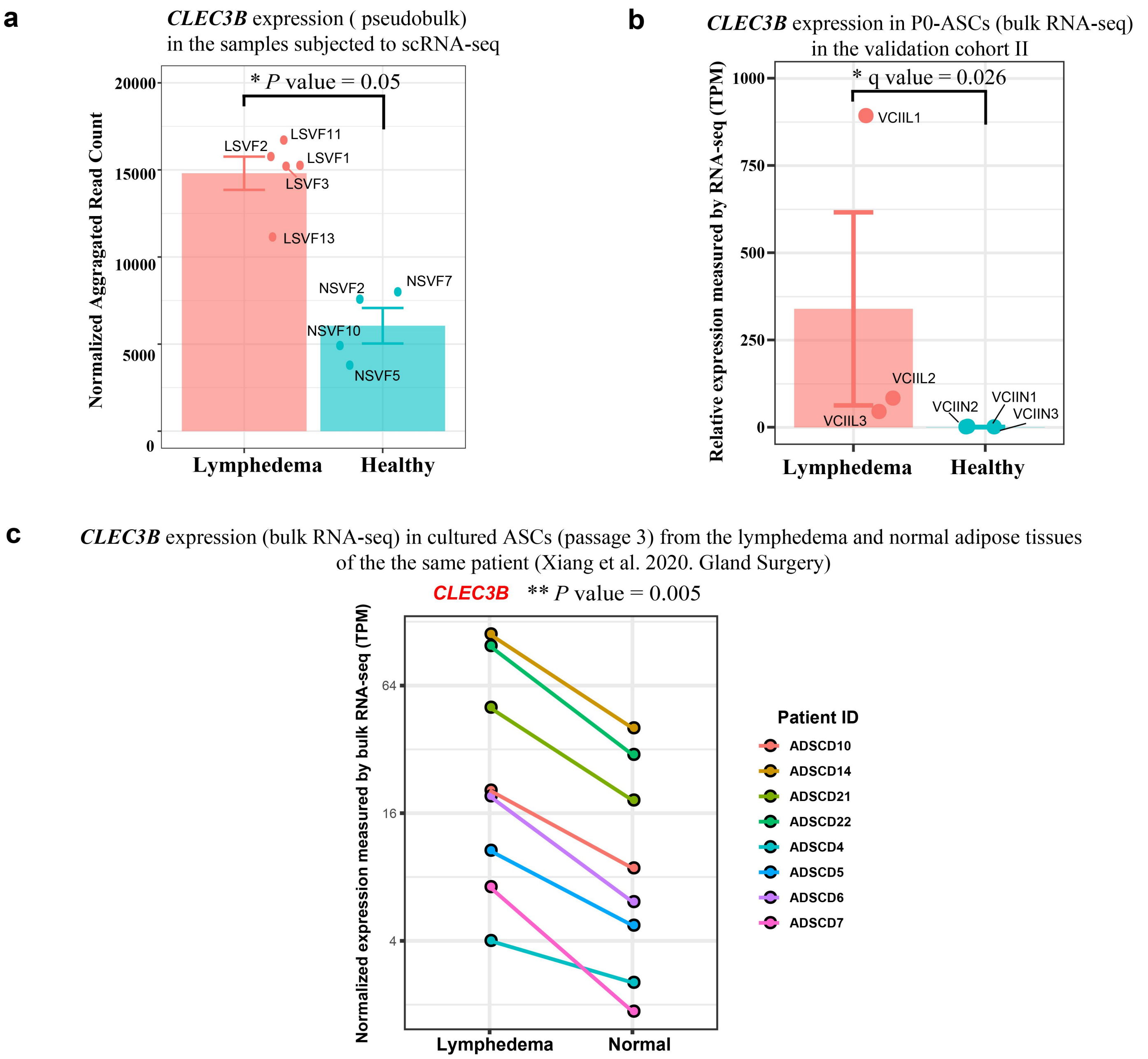

Fig. S13 The elevated expression of *CLEC3B* in ASCs under lymphedema versus healthy conditions in three datasets. a The expression of *CLEC3B* in the samples subjected to scRNA-seq. The expression was determined by aggregating read counts across single cells of ASCs in our scRNA-seq dataset ("pseudobulk"). The differential expression test was performed using a pseudobulk method "edgeR-lrt" implemented in the R package edgeR. *: P value ≤ 0.05. **b** The expression of *CLEC3B* in ASCs freshly isolated from the adipose tissues (P0-ASCs) in the validation cohort II. Age and BMI are matched between the lymphedema (n = 3) and healthy (n = 3) groups (P value > 0.05, Wilcox rank-sum test). The expression was measured by bulk RNA-seq. *: q value < 0.05. Differential expression test implemented in sleuth. **c** The expression of *CLEC3B* in cultured ASCs (passage 3) from the lymphedema and normal adipose tissues of the same patient. The expression was measured by bulk RNA-seq and the date is from the study Xiang et al.'s study (2020, Gland Surgery). ** P value < 0.05, Wilcoxon signed-rank test.

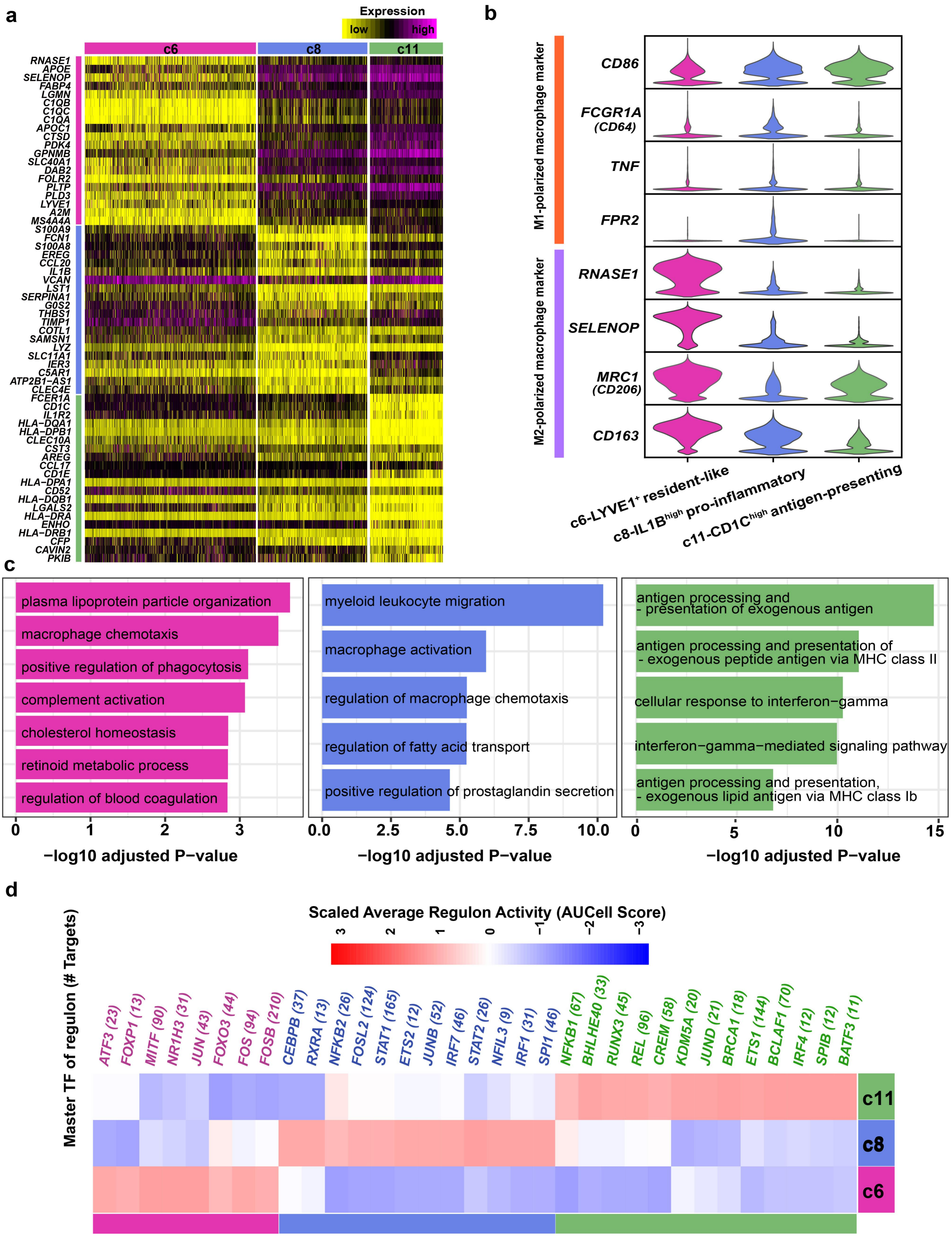

Fig. S14 Adipose tissues of lymphedema display a striking depletion of anti-inflammatory macrophages and a pro-inflammatory microenvironment. a Distinct expression profiles of the three macrophage subpopulations. **b** Expression of M1- or M2-polarized macrophage markers in the three subpopulations. **c** Enriched Gene Ontology terms of the molecular signature for each subpopulation. Adjusted *P* value < 0.05, the hypergeometric test. **d** Subpopulation-specific regulons of each subpopulation detected by SCENIC analysis.

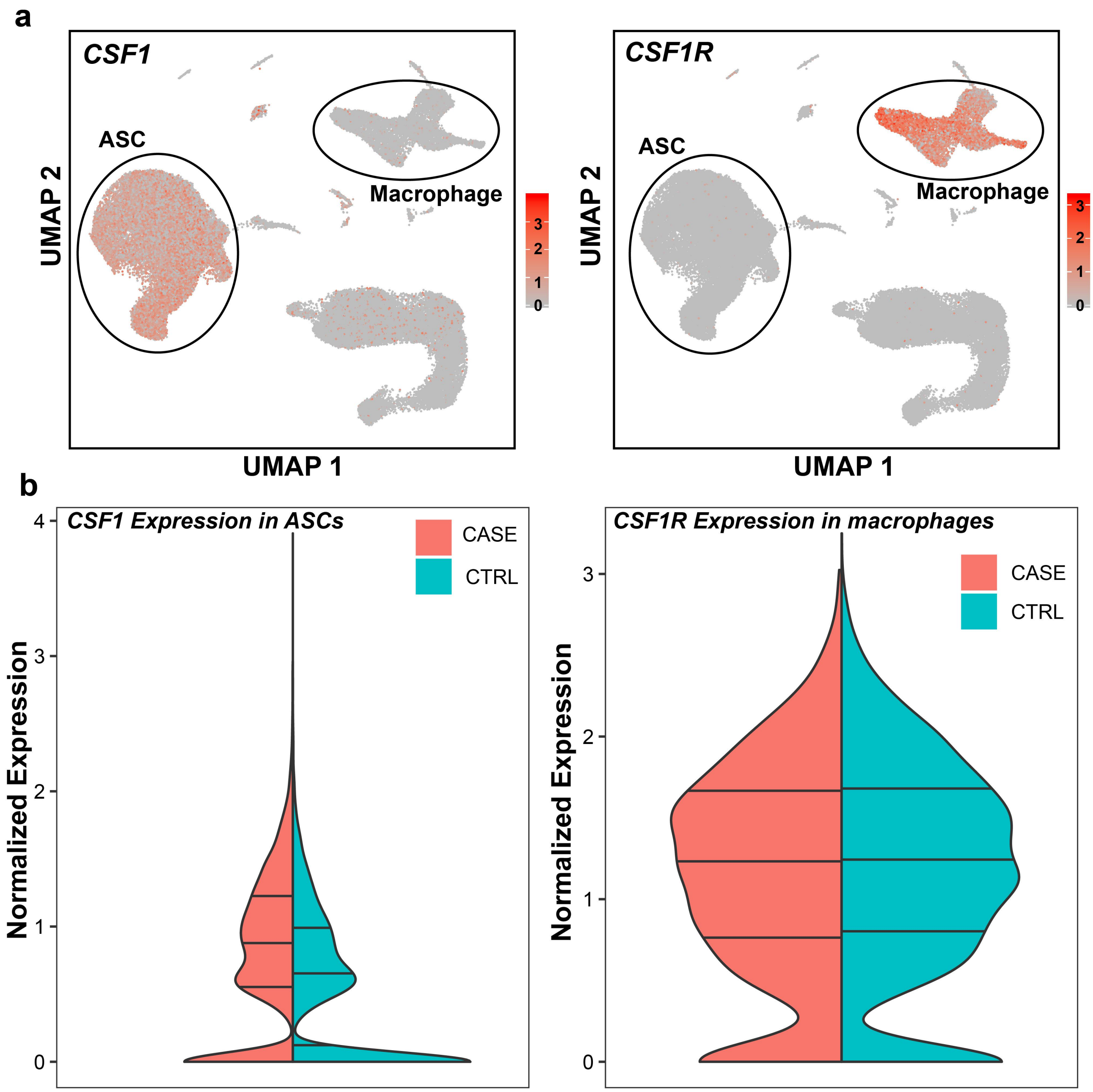

Fig. S15 ASCs are the predominant source of the macrophage colony-stimulating factor CSF1. a ASCs predominately expressed *CSF1* (left panel) and macrophages expressed the receptor *CSF1R* (right panel). **b** The expression of *CSF1* in ASCs (left panel) and *CSF1R* in macrophages (right panel) under CASE (lymphedema) and CTRL (healthy) conditions.
